# Statistical tests for homogeneity of variance for clinical trials and recommendations

**DOI:** 10.1016/j.conctc.2023.101119

**Published:** 2023-03-31

**Authors:** Yuhang Zhou, Yiyang Zhu, Weng Kee Wong

**Affiliations:** Department of Biostatistics, University of California, Los Angeles, CA 90095, United States of America

**Keywords:** Homogeneity of variance, Various tests for clinical trials

## Abstract

In most clinical trials, the main interest is to test whether there are differences in the mean outcomes among the treatment groups. When the outcome is continuous, a common statistical test is a usual t-test for a two-group comparison. For more than 2 groups, an ANOVA setup is used and the test for equality for all groups is based on the F-distribution. A key assumption for these parametric tests is that data are normally, independently distributed and the response variances are equal. The robustness of these tests to the first two assumptions is quite well investigated, but the issues arising from heteroscedasticity are less studied. This paper reviews different methods for ascertaining homogeneity of variance across groups and investigates the consequences of heteroscedasticity on the tests. Simulations based on normal, heavy-tailed, and skewed normal data demonstrate that some of the less known methods, such as the Jackknife or Cochran’s test, are quite effective in detecting differences in the variances.

## 1 Introduction

Given a random variable Y, the variance of Y is $Var\left(Y\right)=E\left[{\left(Y-\mu \right)}^{2}\right]$, where μ is its mean. The variance measures how spread out are its values from the mean. Variance homogeneity is frequently a key assumption for testing equality of means across groups. For example, in clinical trials, the random variable Y is the continuous response from a patient and we wish to test whether there is homogeneity in the response variances across treated groups of patients. A common statistical test for this purpose is the usual t-test for two treated groups or an ANOVA F-test for three or more treated groups. When the variances of responses from different groups are unequal, these tests may no longer be valid and consequently, may not provide the correct statistical inference. In particular, the required control on type 1 and 2 errors become questionable. The extent of invalidity of the test depends on the statistical test itself and how serious the violation of variance homogeneity is. When variances of responses from patients receiving various treatments appear different, a common strategy is to transform the data using a variance stabilizing transformation so that the transformed data is homoscedastic, or nearly so. Sometimes, adjustments are made to the test statistic to accommodate for the non-constant variances. When data transformations are ineffective, alternative statistical tests that do not require variance homogeneity are derived.

There is much research on the validity of the t-test and ANOVA F-test when data are not normally distributed. For example, Knief recently used simulations and showed that the t-test is robust to non-normality and type I error rates over a wide range of conditions [1]. They found that the most serious violation is that of independence and the least serious is that of normality. There are various ways of testing for independence and normality of the data. For instance, to test for normality, statistics based on the kurtosis or skewness of the data may be used [2]. Although no consensus has been made on the extent of non-normality of the data before it becomes problematic [3], parametric tests like t-test and ANOVA F-test should not be applied to data that clearly violate normality assumption and the sample size is small [4]. This is because applying parametric tests to non-normal data can adversely affect the type I error rate [5].

There is quite a bit of work in the literature on the effects of the tests when data are not independent, which is less extensive than those for non-normality. However, some researchers did specify the importance of not violating independence assumptions, especially when using parametric tests. In health care, various analyses require data to be independent, including popular methods in cluster randomized trials tests, like the Chi-squared test and the t-test [6]. Violation of independence assumption not only causes inflation of Type I and Type II errors, but also makes detection of significant differences among treatment groups harder [7]. Further, large effects of violating normality assumption were also found, when variables were non-independent [8]. Accordingly, various new methods of testing independence assumption have also been proposed recently. For example, instrumental variable independence could be tested in the way described by Désiré and Ismael [9]. Local dependence could also be detected as shown by Marieke and Sarah’s work [10]. However, there seems to be little work on investigating heteroscedastic responses in clinical trials and their consequences on statistical tests.

The goal of this paper is to review common and recent tests for homogeneity in the context of a clinical trial and study the consequences when this assumption is invalid. We conduct simulations to investigate the robustness of the various tests to homogeneity and identify tests that seem to be still generally valid under such a violation. Section 2 first reviews tests for homogeneity of variances when there are two treatment groups, before tests for three or more groups are reviewed. In each case, we review parametric tests before non-parametric tests. Section 3 applies some of the tests to a real data set to detect whether there is heteroscedasticity in the 3-treatment group trial and Section 4 conducts a simulation to study the effects of heteroscedasticity on the various tests when data are skewed normal or heavy-tailed. In Section 5, we provide a Shiny app to facilitate tests of homogeneity in a clinical trial when there are 2 or more treatment groups. We conclude in Section 6 with our recommended tests for variance homogeneity based on simulation results and closing remarks.

In what is to follow, we adopt the following notation for the whole paper. We assume that there are k treatments of interest and patients are randomly assigned to one of these treatment groups. The total number of subjects is predetermined and is N. Each treatment group i has $n_{i}$ subjects, and $N=\sum_{i=1}^{k}n_{i}$. When balanced designs are used, we denote the common sample size in each group by n, so $n_{i}=n$ and we have N=kn. Throughout, $Y_{ij}$ denote the jth observation from ith group, $\overset{¯}{Y_{i}}$ is the sample mean response from the ith group, and $\overset{¯}{Y}$ is the sample mean of all observations. Let $s_{i}^{2}$ be the sample variance of ith group, and let $s_{p}^{2}$ be the pooled sample variance from all groups. Similarly, let $\mu_{i}$ be the true mean response from the ith group, and let $\sigma_{i}^{2}$ be the true variance of responses from the ith group.

Throughout the paper, the null hypothesis for all tests is that there is variance homogeneity across the treatment groups versus the alternative that variances from some groups are unequal. The only exception is for Cochran’s test. The notation for the null hypothesis is $H_{0}:\sigma_{1}^{2}=\cdots =\sigma_{k}^{2}$ and the alternative hypothesis is $H_{1}:\sigma_{i}^{2}\neq \sigma_{j}^{2}$ for some 1≤i≠j≤k.

### 1.1 Methods of comparing means

Frequently, the main interest in a clinical trial is to assess treatment efficacy based on mean responses from groups assigned to various treatments. Yet the main decision in selecting an appropriate test is whether we expect group variances to differ.

The t-test and the ANOVA F-test are among the most popular used in practice. However, when data are not normally distributed or heteroscedastic, these tests can become problematic and do not provide the nominal error rates. An alternative is to use the Mann–Whitney U test or a modified version of it [11]. When data have extreme values, one may use the Wilcoxon Signed Rank test to compare the medians from the various treated groups [11].

Recent researches provide more tools in dealing with the question of figuring out if equality of means exists between groups of data. A non-parametric progressive signed rank control chart has been proposed to deal with heavy-tailed or skewed normal data [12].

New methods in testing multivariate means have become another popular area under development. A combination of Hotelling and Simes tests has been proposed as a new method for comparing multivariate mean equality [13]. This new test has the potential to deal with non-equal covariance matrices, and it is robust to violation of the Gaussian assumption. It is worth mentioning that the results of tests of location do depend on the results from tests of homogeneity of variance, especially when the groups for comparison have small sample sizes. To illustrate this, a simple simulation was made. Two samples at size of 15 were simulated from two normal distributions Normal(0,1) and Normal(0,5). This process was repeated 100 times, and the proportions of false rejection by t-test specifying equal or unequal variances were recorded as approximation of type one error.

From the table below, at sample size of 15, the approximation of type one error would increase 50%, from .08 to .12, if one falsely specify equal variances of the two samples. Thus, it is important to both conduct variance homogeneity tests and choose the correct variance homogeneity test before using any location tests (see Table 1).

**Table 1:** Proportion of false rejection by t-test for comparison of means of two samples with sample size = 15 generated from Normal(0,1) and Normal(0,5), out of 100 runs [14], [15]. Specify Equal Variance: If specifying var.equal in t.test() function in R, with Yes = TRUE, No = FALSE. Type one error: proportion of false rejection generated by t-test results, out of 100 runs.

Specify Equal Variance	Type One Error
Yes	0.12
No	0.08

## 2 Methods of comparing variances

There are many different statistical methods to compare variances for two or more groups and for normally distributed or non-normal data. We first review parametric and nonparametric tests for two groups before we describe corresponding tests for more than two treatment groups (see Table 2).

**Table 2:** Summary table of all tests that will be discussed in details in the following sections. All functions are avaliable in RR with corresponding packages at the citation part.[14], [15], [16], [17], [18], [19]. Name: the names of each test. Type: indicating if a test is parametric or non-parametric. Function: the specific function of each test in R. *:Levene’s test has its variances in different forms. There are both parametric form and non-parametric form to Levene’s tests.

Test Name	Type	Function
F-test	Parametric	var.test()
Ansari-Bradley test	Non-Parametric	ansari.test()
Moses Rank-liked test	Non-Parametric	moses.test()
Jackknife test	Non-Parametric	miller.jack()
Levene’s test	Both*	levene.test()
Bartlett’s test	Parametric	bartlett.test()
Hartley’s test	Parametric	hartley.test()
Cochran’s test	Parametric	C.test()
Brown-Forsythe test	Non-Parametric	oneway.test()
Fligner-Killeen test	Non-Parametric	fligner.test()

### 2.1 Two-sample tests

#### 2.1.1 Parametric tests

##### 2.1.1.1 F-test

This is the most common test for variance homogeneity for randomized studies with two groups of sizes $N_{1}$ and $N_{2}$ when the data are normally and independently distributed. The test statistic is the ratio of the two sample variances $F=s_{1}^{2}/s_{2}^{2}$, and we compare the value of the F statistic to an upper percentile of the F-distribution with degrees of freedom $n_{1}-1$ and $n_{2}-1$. Since the test statistic uses sample variances, this test can be sensitive to outliers and non-normality. For an α-sized one-sided alternative, such as, $\sigma_{1}^{2}<\sigma_{2}^{2}$, we reject the null hypothesis in favor of the alternative when $F<F_{\alpha ,N_{1}-1,N_{2}-1}$. For testing an α-sized two-sided alternative of $\sigma_{1}^{2}\neq \sigma_{2}^{2}$, we reject the null hypothesis in favor of the alternative when $F>F_{\alpha }/2,n_{1}-1,n_{2}-1$ or $F<F_{1-\alpha /2,n_{1}-1,n_{2}-1}$.

The above F-test for the null hypothesis is easily computed using any statistical package or on an Excel spreadsheet. For instance in R, one may use the function var.test and specify the confidence level sought and the hypothetical value of $\sigma_{1}^{2}/\sigma_{2}^{2}$ to test for. Usually, the interest is in testing whether the ratio is equal to unity versus not in the alternative hypothesis, which may be one-sided or two-sided. In STATA one may simply use a similar command sdtest with appropriate options for controlling the type 1 error rate. For example, if data is arranged in a long format, and we want to test whether variability in the length of stay at hospitals by gender is equal, the command “sdtest length, by(sex) level(80)” will compute the test at the 80% confidence level.

We note that tests that assume variance homogeneity in the data can pose significant theoretical challenges when the assumption is violated. For example, the well-known Behrens–Fisher problem tests the equality of means of two normal populations with different variances using two independent samples. One common approach is to apply a Welch t-test based on the argument that under the null hypothesis,

$$\frac{\left(\overset{¯}{Y_{1}}-\overset{¯}{Y_{2}}\right)-\left(\mu_{1}-\mu_{2}\right)}{\sqrt{\frac{s_{1}^{2}}{n_{1}}+\frac{s_{2}^{2}}{n_{2}}}}$$

has approximately a t-distribution with degrees of freedom equal to

$$df=\frac{{\left[\frac{s_{1}^{2}}{n_{1}}+\frac{s_{2}^{2}}{n_{2}}\right]}^{2}}{\frac{{\left(\frac{s_{1}^{2}}{n1}\right)}^{2}}{n_{1}-1}+\frac{{\left(\frac{s_{2}^{2}}{n_{2}}\right)}^{2}}{n_{2}-1}}.$$

The complicated expression for the degrees of freedom is obtained by the method of moments described in Satterwaithe [20]. The Welch t-test is implemented in STATA by specifying “unequal” or “welch” as an option in the ttest command. In R, the user specifies var.equal=FALSE as an option.

There is a research on the Behrens–Fisher problem and Dudewicz et al. that provides a good review [21]. The authors also developed an exact and optimal solution to the Behrens–Fisher problem, where they used a two-stage approach and an additional parameter c to control the power of the test [21]. Extensions to testing equality of means from multivariate normal distributions under heteroscedasticity are also available. For example, Eftekhar constructed a fiducial test by inverting the fiducial confidence regions of differences between normal mean vectors [22].

#### 2.1.2 Non-parametric tests

Non-parametric methods, known as “distribution-free methods”, require fewer assumptions than parametric methods. It does not mean that the methods require no assumption on the distributions of the underlying data. We first review some non-parametric methods for comparing variances between two treated groups.

##### 2.1.2.1 Ansari–bradley test [23]

With equal medians, two independent samples are assumed to come from densities of the form f((t−m)/γ) and f(t−m), where m is an unknown nuisance parameter and γ, the ratio of scales, is the parameter of interest. Setting θ as the ratio of the variances from the two groups, the Ansari–Bradley test evaluates the null hypothesis that γ=1 and the alternative hypothesis can be either γ>1, γ<1 or γ≠1..

Like other non-parametric tests, this Ansari–Bradley test is rank based with a unique ranking scheme. Suppose there are $n_{i}$ observations from group i,i=1,2 and, without loss of generality, assume that $n_{1}<n_{2}$. First, rank all observations from both groups from smallest to largest; then rank the smallest and the largest as “1”, and second smallest and second largest as “2”, and so on. In this ranking scheme, observations closer to the median will have larger ranks, and observations far away from median will have smaller ranks. Under the null hypothesis of equal dispersion (with equal median assumed), any $n_{1}$ out of the $n_{1}+n_{2}$ observations will have equal chance of being from group 1, thus we have a bell-shaped discrete distribution of sum of ranks W for group 1 under the null hypothesis. The rejection region will be either at one end or both ends of the bell-shaped curve, depending on the alternative hypothesis. Large sample approximation can also be performed using mean and variance of this distribution.

The table below is from the original published paper in 1960 that displays critical values for the W statistic for both upper and lower tails in different scenarios (see Table 3, Table 4, Table 5).

When the medians of the two groups are unequal, the Ansari–Bradley test is not valid. To fix this problem one can manually make the two medians equal by estimating the medians of both groups and shifting all the data points accordingly. A common way to estimate the median is to find the median of Walsh’s means. We recall that Walsh means are the means of any 2 observations (with replacement). As an example, if we have 5 observations, $Y_{1},Y_{2},Y_{3},Y_{4},Y_{5}$, the Walsh means are shown in Table 5.

**Table 3:** Lower and upper significance levels of W(1). m and n are sample sizes of the two group [23]. The numbers from .995 to .005 are significant levels. Only m = 2 are presented here.

m	n	.995	.99	.975	.95	.05	.025	.01	.005
2	5	–	–	–	2	–	–	–	–
2	6	–	–	–	2	8	–	–	–
2	7	–	–	–	2	9	–	–	–
2	8	–	–	2	2	10	10	–	–
2	9	–	–	2	2	11	11	–	–
2	10	–	–	2	2	12	12	–	–
2	11	–	–	2	2	13	13	–	–
2	12	–	–	2	2	14	14	–	–
2	13	–	2	2	2	14	15	–	–
2	14	–	2	2	2	15	16	16	–
2	15	–	2	2	2	16	17	–	–
2	16	–	2	2	2	17	17	18	–
2	17	–	2	2	2	18	19	–	–
2	18	–	2	2	2	19	19	20	–

**Table 4:** Lower and upper significance levels of W(2). m and n are sample sizes of the two group [23]. The numbers from .995 to .005 are significant levels. Only m = 5 are presented here.

m	n	.995	.99	.975	.95	.05	.025	.01	.005
5	5	–	9	10	10	20	20	21	–
5	6	9	9	10	11	22	23	24	24
5	7	9	10	11	11	24	24	25	26
5	8	10	10	11	12	26	26	28	29
5	9	10	11	12	13	27	28	29	30
5	10	10	11	12	14	29	30	32	32
5	11	11	12	13	14	31	32	33	34
5	12	11	12	14	15	33	34	36	37
5	13	11	13	14	16	34	36	37	38
5	14	12	13	15	16	36	38	40	41
5	15	12	14	15	17	38	40	41	43

**Table 5:** The n(n+1)/2 Walsh means for a sample size of n observations and n=5[23].

--	$Y_{1}$	$Y_{2}$	$Y_{3}$	$Y_{4}$	$Y_{5}$
$Y_{1}$	$Y_{1}$	$\frac{Y_{1}+Y_{2}}{2}$	$\frac{Y_{1}+Y_{3}}{2}$	$\frac{Y_{1}+Y_{4}}{2}$	$\frac{Y_{1}+Y_{5}}{2}$
$Y_{2}$	–	$Y_{2}$	$\frac{Y_{2}+Y_{3}}{2}$	$\frac{Y_{2}+Y_{4}}{2}$	$\frac{Y_{2}+Y_{5}}{2}$
$Y_{3}$	–	–	$Y_{3}$	$\frac{Y_{3}+Y_{4}}{2}$	$\frac{Y_{3}+Y_{5}}{2}$
$Y_{4}$	–	–	–	$Y_{4}$	$\frac{Y_{4}+Y_{5}}{2}$
$Y_{5}$	–	–	–	–	$Y_{5}$

However, some statisticians argue that manipulating medians is not a distribution-free practice. With such concerns, one may consider other methods introduced below. Nevertheless, this test can be carried out in R using the function ansari.test by specifying the two samples, alternative hypothesis, using large sample approximation or not, and the confidence level.

##### 2.1.2.2 Moses rank-like test [24]

Another test for evaluating equality of variances from different groups is the Moses rank-like test developed by Moses [24]. Assumptions for this test are similar to Ansari–Bradley Test except that medians are now unequal and unknown. The test proceeds as follows:

1. Divide observations in the 2 groups into subsets of equal size k; discard additional observations;

2. Calculate $D_{i}=\sum_{j=1}^{n_{i}}{\left(X_{ij}-\overset{¯}{X_{i}}\right)}^{2}$, the sum of squares for the ith subset;

3. Perform a Wilcoxon’s Rank Sum Test on the two groups of D’s.

Test results may vary depending on the division of the observations. A problem with this approach is that the group membership may be manipulated to achieve certain results. This test was developed at a time when computing power was limited, and it is now highly recommended to do this test repeatedly, such as using bootstrap.

##### 2.1.2.3 Jackknife test [25]

The assumptions for this test are that observations from the two groups A and B are independent and they come from continuous distributions with finite 4th moment (Kurtosis). Assume group A has sample size $n_{1}$ and group B has sample size $n_{2}$. If the goal is to estimate a parameter, it does so by systematically leaving out each observation from the data set and calculating the estimate, and then finding the average of these calculations. The procedure for the test is as follows:

1. Find the leave-one-out sample variance for group A, marked as $D_{\left(i\right)},i=1,2,\ldots ,n_{1}$. Denote the sample variance for group A by $D_{0}$

2. Let $S\left(i\right)=ln\left(D_{i}\right)$, let $S_{\left(0\right)}=ln\left(D_{\left(0\right)}\right)$, and let $A_{i}=n_{1}S_{\left(0\right)}-\left(n_{1}-1\right)S_{\left(i\right)}$.

3. Let $\overset{¯}{A}=\sum_{i=1}^{n_{1}}\frac{A_{i}}{n_{1}}$ and let $V_{A}=\sum_{i=1}^{n_{1}}\frac{{\left(A_{i}-\overset{¯}{A}\right)}^{2}}{n_{1}\left(n_{1}-1\right)}$

4. Repeat the above procedure for Group B, and obtain $\overset{¯}{B}$ and $V_{B}$

5. The test statistic for the null hypothesis is $Q=\frac{\overset{¯}{B}-\overset{¯}{A}}{\sqrt{V_{A}+V_{B}}}$ and under the null hypothesis, Q is approximately standard normal, or to be more exact, is distributed as a t distribution with $n_{1}+n_{2}-2$ degrees of freedom. This test can also give us an estimate of the ratio of two group’s variances, $\overset{ˆ}{\gamma^{2}}=e^{\overset{¯}{B}-\overset{¯}{A}}$.

To perform this test in R, one may use the function miller.jack in the package nonpar. For non-parametric two sample location comparison, unequal variances may reduce robustness of the Wilcoxon Rank Sum Test and is thus not recommended. We suggest the Fligner–Policello location test, which is a robust version of Mann–Whitney U test.

### 2.2 Multi-sample tests

There are clinical trials where patients are randomized to more than two treatment arms. We now discuss tests to compare variances of observations from multiple groups. Unless otherwise specified, the null hypothesis is equal variances across all groups and the alternative hypothesis is that variances are not all equal across the groups. All tests are performed at α level.

#### 2.2.1 Parametric tests

##### 2.2.1.1 Levene’s test [26]

This test was developed by Levene and the test assumes that data $Y_{ij}$’s are independent and normally distributed. The test does not depend on the sample variances and so it is not very sensitive to outliers.

Using the notation in Section 1, let $Z_{ij}=\left|Y_{ij}-{\overset{¯}{Y}}_{i}\right|$, let $\overset{¯}{Z_{..}}=\sum_{i=1}^{k}\sum_{j=1}^{n_{i}}Z_{ij}/N$ and let $\overset{¯}{Z_{i.}}=\sum_{j=1}^{n_{i}}Z_{ij}/N_{i}$ for group i. The test statistic for variance homogeneity across groups is

$$W=\frac{\left(N-k\right)}{k-1}\frac{\overset{k}{\underset{i=k}{\sum }}n_{i}{\left(Z_{i.}-Z_{..}\right)}^{2}}{\overset{k}{\underset{i=k}{\sum }}\overset{n_{i}}{\underset{j=1}{\sum }}{\left(Z_{ij}-Z_{i.}\right)}^{2}}$$

which under the null hypothesis, has a F-distribution with numerator degree of freedom k−1 and denominator degree of freedom N−k. At the α level of significance, we reject the null hypothesis if $W>F_{\alpha ,k-1,N-k}$.

The test statistic has a very similar form to the F-test in ANOVA setting if we re-write the test statistic as

$$W=\frac{\frac{\overset{k}{\underset{i=1}{\sum }}n_{i}{\left(Z_{i.}-Z_{..}\right)}^{2}}{k-1}}{\frac{\overset{k}{\underset{i=1}{\sum }}\overset{n_{i}}{\underset{j=1}{\sum }}{\left(Z_{ij}-Z_{i.}\right)}^{2}}{N-k}},$$

where the numerator is the between group mean sum of squares of Z and the denominator is the within group mean sum of squares of Z. Similar to the ANOVA situation, the W test statistic is also compared to the F distribution.

To perform the test in STATA, one may either use the command robvar, and the $w_{0}$ statistic in the output gives results of Levene’s test. In R, one may use the levene.test function in the package lawstat, and specify location = “ mean” to perform a Levene’s test.

##### 2.2.1.2 Bartlett’s test [27]

The test statistic of Bartlett’s test is [27]:

$$\chi^{2}=\frac{\left(N-k\right)\;\text{ln}\;\left(S_{p}^{2}\right)-\overset{k}{\underset{i=i}{\sum }}\left(n_{i}-1\right)ln\left(S_{i}^{2}\right)}{1+\frac{1}{3\left(k-1\right)}\left(\overset{k}{\underset{i=1}{\sum }}\left(\frac{1}{n_{1}-1}\right)-\frac{1}{N-k}\right)}.$$

The rationale of this test can be seen as rewriting the denominator of this test statistic as $\sum_{i=1}^{k}\left(n_{i}-1\right)\left(ln\left(S_{p}^{2}/s_{i}^{2}\right)\right)$, which is the sum of log ratio of pooled sample variance and each group’s sample variance, weighted by each group’s sample size minus 1.

**Fig. 1:**
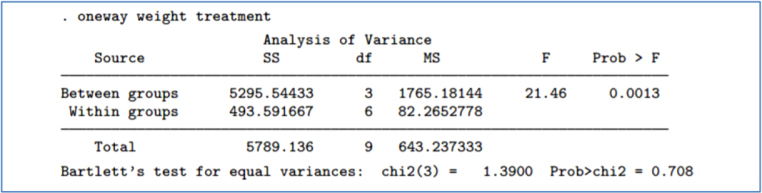
Results in the STATA output from the one-way command contains the Bartlett’s test result [28].

In R, one may obtain the test result using the command bartlett.test. In STATA, the result for Bartlett’s test for equal variance will be automatically displayed in the output after the one-way ANOVA command Here is an example, where “weight” is the continuous outcome and “treatment” designates group membership (see Fig. 1).

##### 2.2.1.3 Hartley’s test [29]

Hartley [29] proposed a test to ascertain equality of variances across groups in a randomized trial. It requires that data are independent, normally distributed and the sample size in each group is equal. The test statistic is the ratio of the largest group variance to the smallest group variance.

$$F_{max}=\frac{\text{estimated largest group variance}}{\text{estimated smallest group variance}}.$$

Under the null hypothesis, the value of the test statistic $F_{max}$ is compared to a critical value in a special $F_{max}$ table, which depends on the number of treatments and the degree of freedom, which is the common sample size in each group minus 1. If $F_{max}$ is smaller than the critical value, we conclude homogeneity; otherwise, we conclude non-homogeneity.

The table below lists critical values of the Hartley’s test statistic $F_{max}$ for different sample sizes and type 1 error rates at α=0.05 and α=0.01 level. When the sample size for each group goes to infinity, meaning that the sample variance for each group is the true variance, the critical value will be 1. The R package SuppDists has a distribution called maxFratio, and provides the critical values by specifying the number of groups and the common sample size for each group.

Jesse (2010) provided an algorithm to find critical values of Hartley’s test and demonstrated the possibility of applying Hartley’s test to an unbalanced design [30] (see Table 6).

##### 2.2.1.4 Cochran’s C test [31]

This test is among the earliest and was proposed by Cochran to test whether variance from one group is relatively large compared with other groups; so in some sense, it is an outlier test. The assumptions for the tests are that data are independent and normally distributed and all groups have equal size. The idea of this test is to compare the variance of one group to all the other groups [31]. Unlike other tests we have discussed above, this test detects one exceptionally large variance value at a time and does not test for overall homogeneity.

**Table 6:** Critical values of $f_{max}$ for Hartley’s homogeneity of variance test [30]. The value 2 to 5 on the top are the number of treatments. The number 2 to 5 on the left are degrees of freedom. The upper value for a specific treatment and a specific degrees of freedom is for α=.05, and the lower value is for α=.01. For the unequal sample size, use the smaller of the degrees of freedom for the two variances being compared.

	2	3	4	5	...
2	39.0	87.5	142	202	...
	199	448	729	1036	...
3	15.4	27.8	39.2	50.7	...
	47.5	85.0	120	151	...
4	9.6	15.5	20.6	25.2	...
	23.2	37.0	49.0	59	...
5	7.2	10.0	13.7	16.3	...
...	14.9	22.0	28.0	33	...

The test statistic is the sample variance of one group divided by the sum of the sample variances from all groups, i.e.

$$C_{j}=\frac{s_{j}^{2}}{\overset{k}{\underset{i=1}{\sum }}s_{i}^{2}}.$$

If N is the total sample size and n is the common group size, the critical value for the above one-sided α-sized test is [32]

$$C_{UL}\left(\alpha ,n,N\right)=1+\frac{N-1}{F\left(\frac{\alpha }{N},\left(n-1\right),\left(N-1\right)\left(n-1\right)\right)}.$$

If the test statistic $C_{j}$ exceeds this upper bound, we conclude that the variance for group j is significantly larger than other groups. The code for Cochran’s C test is available in the R package GAD and provides the test result easily using the command C.test.

When there may be unequal variances among multiple groups, Welch’s ANOVA test is usually used instead of the standard ANOVA test for making inferences on the means of the groups. Following notation in Section 1, let $w_{i}=n_{i}/s_{i}^{2}$ be the “weight” for the ith group, let $w=\sum_{i=1}^{k}w_{i}$, and let ${\overset{¯}{Y}}^{\prime }=\sum_{i=1}w_{i}{\overset{¯}{Y}}_{i}/w$. Then the test statistic for Welch’s ANOVA is

$$F=\frac{\frac{1}{k-1}\overset{k}{\underset{i=1}{\sum }}w_{i}{\left({\overset{¯}{Y}}_{i.}-{\overset{¯}{Y}}^{\prime }\right)}^{2}}{1+\frac{2\left(k-2\right)}{k^{2}-1}\overset{k}{\underset{i=1}{\sum }}\left(\frac{1}{n_{i}-1}\right){\left(1-\frac{w_{i}}{w}\right)}^{2}}.$$

Under the null hypothesis of equal means, [33] showed that this statistic has a F(k-1, df) distribution where df is

$$df=\frac{k^{2}-1}{3\overset{k}{\underset{j=1}{\sum }}\left(\frac{1}{n_{j}-1}\right){\left(1-\frac{w_{j}}{w}\right)}^{2}}.$$

To perform this test in STATA, one may use the function fstar or wtest; in R, one may specify var.equal=FALSE as an option in the one-way ANOVA command oneway.test.

#### 2.2.2 Non-parametric tests

##### 2.2.2.1 Brown-Forsythe test [34]

Brown-Forsythe test is essentially Levene’s test using medians instead of means from the various groups. In this case $Z_{ij}=\left|Y_{ij}-{\overset{˜}{Y}}_{i.}\right|$, where ${\overset{˜}{Y}}^{i.}$ is the median of ith group. In STATA, the w50 statistic in the output of Levene’s test gives the result of the Brown-Forsythe test; in R, one may use the levene.test function in the package lawstat, and specify location=“median” as an option.

To adjust for non-normal data, one can also use trimmed means when performing Levene’s test. In this case, we use $Z_{ij}=\left|Y_{ij}-{\overset{¯}{Y}}_{i.}^{\prime }\right|$ instead, where ${\overset{¯}{Y}}_{i.}^{\prime }$ is the trimmed mean of observations from the ith group after excluding the upper and lower extreme values For example, 25% of the observations in the upper and lower tail ends of the data may be excluded when the trimmed mean is computed.

##### 2.2.2.2 Variations of Levene’s test [35]

Nordstokke and Zumbo developed a non-parametric Levene’s test, where ranks of all observations are used instead of the original values [35]. They showed that their test is generally more robust than other tests under the null hypothesis. In R, one may generate a rank variable for all observations first before Levene’s test on the ranks is performed.

##### 2.2.2.3 Fligner–Killeen test [36]

Fligner–Killeen test is a test for equality of variance among multiple groups, and is believed to be robust to the normality assumption. We first rank $\left|Y_{i,j}-{\overset{˜}{Y}}_{i}\right|$ where ${\overset{˜}{Y}}_{i}$ is the median for ith group. Then we assign increasing scores to each rank m, given by

$$a_{N,m}=\Phi^{-1}\left(\frac{1+\frac{m}{N+1}}{2}\right),$$

using the inverse normal distribution $\Phi^{-1}$. We next define the mean increasing score for group i by

$${\overset{¯}{A}}_{i}=\frac{1}{n_{i}}\overset{n_{i}}{\underset{i=1}{\sum }}a_{N,m_{i,j}},$$

where $a_{N,m_{ij}}$ is the increasing rank score for jth observation in the ith group. Let the overall mean increasing score be

$$\overset{¯}{a}=\frac{1}{N}\overset{N}{\underset{m=1}{\sum }}a_{N,m},$$

and let

$$V^{2}=\frac{1}{N-1}\overset{N}{\underset{m=1}{\sum }}{\left(a_{N,m}-\overset{¯}{a}\right)}^{2}.$$

The test statistic is

$$x_{0}^{2}=\frac{\overset{k}{\underset{i=1}{\sum }}n_{i}{\left({\overset{¯}{A}}_{i}-\overset{¯}{a}\right)}^{2}}{V^{2}}$$

and under the null hypothesis of equal variances across all groups, $x_{o}^{2}$ has a $\chi^{2}$ distribution with k-1 degrees of freedom.

Since all data points are ranked by their closeness to the median and the rank is mapped to a normal density, extreme values would not affect the test statistics and therefore the test is robust against non-normality. In R, results from this test can be obtained using the function fligner.test.

For non-parametric multiple sample location comparison, one may use Kruskal–Wallis test to compare mean ranks instead of medians.

## 3 Clinical applications

We use a clinical data set called “coagulation” from the R package SimComp. The description of the data set reads “three sets of extracorporeal circulation in heart-lung machines: treatments H and B, and standard S. Twelve (S and H each) and eleven (B) male adult patients were enrolled in the trial. The analysis is based on a set of laboratory parameters restricted to the blood coagulation system, characterized by three primary endpoints (each as quotient from post- and pre-surgery values). Higher values indicate a better treatment effect. For more details on this study, see Kropf et al. (2000)” (see Table 7).

We want to compare the mean response for the three endpoints (Thromb.count, ADP, TRAP) among the 3 treated groups. Preliminary examinations show that the distributions of Thromb.count and ADP are quite normal for each group. Given this information, we accordingly choose our methods to test the equality of variances in the 3 groups (see Table 8).

**Table 7:** A sample of observations from the “coagulation” data set [37].

Patient	Thromb.count	ADP	TRAP	Group
7	1.0456323	0.97796	1.3744736	B
8	0.8512342	0.8992643	0.4320755	H
9	1.2339782	1.1099057	0.580081	H
10	1.2443439	1.2429597	0.7925148	B
11	0.8874788	0.9132075	0.5672504	B
12	0.8578994	0.8609023	0.8244653	S
19	0.7236927	0.7753389	0.4151449	S

The variables Thromb.count and ADP seem to be normally distributed, thus we can also do Bartlett’s test for these two variables. The table below shows that results from Bartlett’s test give the same conclusions as Levene’s test (see Table 9).

**Table 8:** Test for equal variance for the 3 endpoints [14].

Variable	Test applied	p-value	Conclusion
Thromb.count	Levene’s test	0.2499	Cannot reject equal variance null hypothesis
ADP	Levene’s test	0.0259	Variance not all equal
TRAP	Brown-Forsythe test	0.9922	Cannot reject equal variance null hypothesis

Now that we have verified the heterogeneity for the ADP variable, we know that we should use Welch’s ANOVA to make inferences. Without assuming equality of variance, Welch’s ANOVA on ADP versus treatment groups gives a p-value of 0.0452; but when the equality of variance is assumed, ANOVA on ADP versus treatment groups gives a p-value of 0.05312. In this particular case, a significant result could be dismissed if the researchers applied the wrong test.

**Table 9:** Bartlett’s test for equal variance [14].

Variable	Test applied	p-value	Conclusion
Thromb.count	Bartlett’s test	0.2264	Cannot reject equal variance null hypothesis
ADP	Bartlett’s test	0.0058	Variance not all equal

## 4 Simulations

### Objective of the simulations

The goal of the simulations is to figure out the test that can best detect the difference in variances when the two samples consist of the same sample size, from the same population, and only differ in their variances. We also aim to investigate which tests are more likely to detect that difference in simulated data from different kinds of distributions (normal, skewed normal, heavy-tailed), in different variance differences between or among populations, or in different sample sizes.

### Design of the simulations

The whole process is conducted using R Studio. Three types of distributions of populations are involved in this process including normal distribution, t-distribution, and skewed normal distribution. The following sections would elaborate in detail on how parameters are chosen for each specific distribution.

For two-sample tests, one run includes generating two sets of random numbers of a specific sample size from two specific distributions respectively that only differ in variance. After 2000 runs, there would be 2000 pairs of data. Then, different tests described in Section 2 would be applied to each pair of the data, and the number of times that a test successfully detects a variance difference between a pair would be recorded for each test. A higher number would indicate that the specific test is more likely to detect a variance difference (at .05 critical region) under a specific sample size, distribution, and variance difference. The variance difference is indicated by their ratios, with 1:1 indicating two distributions have the same variance. The procedure of 2000 simulation runs and applying different tests to each of them are repeated for variance ratios of 1:1.5, 1:2, 1:4, and 1:9 when data are simulated from normal or skewed normal distributions. Note that we could not directly simulate t-distribution with the same mean but different variance ratios. Pairs of data simulated from t-distributed populations have degrees of freedom of 3:12, 3:6, and 3:4; respectively, they would have variance ratios of 5:2, 2:1, and 3:2. Since the function used to simulate data from t distributions set the center location of 0 by default, we generated pairs of data come from almost same heavy-tailed populations with the same mean but different variances. Then, for each unique distribution, with a specific variance ratio, the 2000 simulation runs are done repeatedly for a sample size ranging from 11 to 100.

For multi-sample tests, the procedure is pretty similar to the two sample ones with some small changes. Note that during each run this time, only three sets of random numbers are generated to find out a test’s ability to detect variance differences among multiple groups because the whole simulation process would take too much time. Since now we have three sets of data, the variance ratios of them become 1:1:1.5, 1:1:2, 1:1:4, and 1:1:9 for normal and skewed normal distributions. For heavy-tailed distributions, the ratios of degrees of freedom are 3:12:12, 3:6:6, and 3:4:4, with variance ratios 5:2:2, 2:1:1, and 3:2:2, respectively.

Despite variance ratios, for data generated from heavy-tailed distributions, we further examine if different tests would detect the variance difference when the heaviness of tails changes. The shape parameter in the function we used to generate data from heavy-tailed distributions would help us to change the heaviness of their tails. We consider shape = 10 and shape = 50 to present separate results.

The results would be presented in plots with the x-axis indicating the sample sizes data generated from a specific distribution and the y-axis indicating the proportion of correct rejection, as an approximation of power, out of 2000 runs. Section 4.1.1 would give a detailed explanation of one plot, and the remaining sections in Section 4 would present critical results from simulations.

Before final decision on which tests are suitable in each scenario, plot of the proportion of false rejection would also be presented as an approximation of type one errors of different tests. The design of the simulations that generate these information are pretty similar to the design described in the previous paragraphs, except that now both mean and variance are equal for each pair of samples. Note that for t distributions, all samples would be simulated from t distribution with a degrees of freedom of three to control for variance. Based on both power and error approximation, a summary table would be provided at Section 4.4 to illustrate what tests should be used in different scenario.

**Fig. 2:**
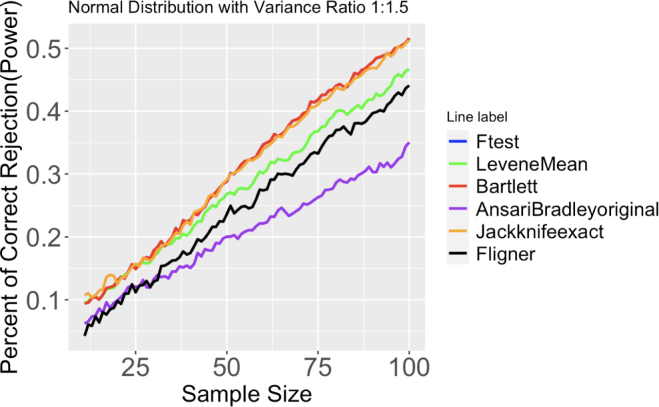
Proportion of correct rejection, or power, of different tests out of 2000 runs. Two normal distributions have same means, but differ in variance ratio(1:1.5). Sample size ranges from 11 to 100.

To make a easy visualization, all plots would only report the test with largest power or lowest error for the variation of a specific test. For example, four variations of Levene’s tests are performed during the simulation, Levene’s test using trimmed mean, mean, median, and non-parametric method. In Fig. 2, only the result of Levene’s test using mean would be reported, since it has relatively higher power compared with other forms of Levene’s test.

### 4.1 Two-sample simulations

For the two-sample scenario, there are 11 tests, including multi-sample methods and non-parametric methods: F test, Levene’s test using the sample mean, Levene’s test using sample median, Levene’s test using trimmed mean, non-parametric Levene’s test, Bartlett’s test, Ansari–Bradley test without median adjustment, Ansari–Bradley test with median adjustment, Jackknife using normal approximation, Jackknife using exact t-distribution, and Fligner–Killeen test. Levene’s test using trimmed mean ignored the upper 25 percent and lower 25 percent of data when calculating the mean. For a given sample size, test results yielding the best rejection rates are tests that have the largest value on the y-axis in the figures. Note that approximate Jackknife and exact Jackknife are essentially the same tests using different rejection rules.

#### 4.1.1 Data with normal distribution

The x-axis in Fig. 6 is the sample size of two sets of data simulated from two normal distributions with the same mean but different variances. The variance ratio of the two distributions here is 1:1.5. The y-axis indicates the proportion of correct rejection out of 2000 runs as an estimation of power of each test. The higher this number, the more powerful the test is in terms of detecting variance heterogeneity. For example, with a sample size of 50, the estimated power of F-test is .288 out of a total of 2000 runs. On the other hand, the power of Fligner test is only .201. This means F-test is a more powerful test in terms of detecting variance differences at this point.

The following paragraphs discuss simulation results for the cases when there are large differences in the variances and the sample sizes are larger than 50 approximately. They are based on observations from Figures 2, 12, 13, 14 and also Figures numbered larger than 14 which can be found in the supplemental materials.

For both large variance ratios (1:9) and large sample sizes (n larger than 50), almost all tests reject the null hypothesis of equal variance for all iterations. As either sample size or variance ratio decreases, F-test, Bartlett’s test, and Jackknife test become more powerful than all other tests. In an extreme situation of both small sample size (n smaller than 20) and small variance ratio (1:1.5), Levene’s test using mean as the parameter becomes comparably powerful compared with the other three.

#### 4.1.2 Data with heavy tailed(T) distribution

**Fig. 3:**
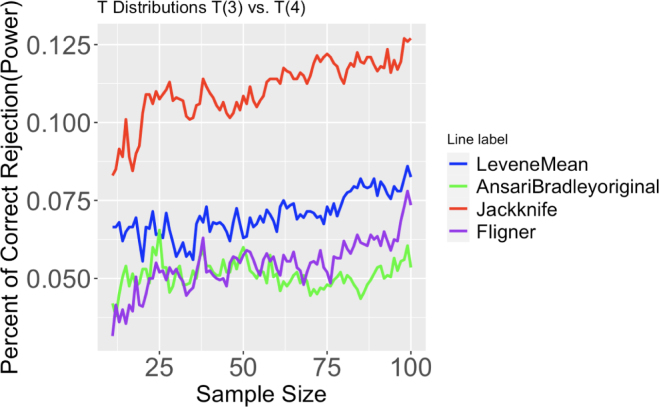
Proportion of correct rejection, or power, of different tests out of 2000 runs. Two T distributions have degrees of freedom 3 and 4. Sample size ranges from 11 to 100.

The variance for the t distribution with v degrees of freedom, denoted by t(v) is $\frac{v}{\left(v-2\right)}$ if v>2. This means that the variance for t(3) is 3, for t(12) it is 1.2, for t(6) it is 1.5, and for t(4) it is 2. By comparing two data sets from two different t distributions, we can simulate the scenario where tails are heavy and variances are unequal. Fig. 3, 15, and 16 support findings in the following paragraphs.

For such distributions, the F-test and Bartlett’s test could not be applied because the distribution of data is not normal. However, Levene’s tests are still valid since the assumption is approximate normality. Results show that the Jackknife method has always been the best test to identify differences in variances across groups and sample sizes. When the sample size is small, the exact Jackknife method does better than the approximate Jackknife.

#### 4.1.3 Data with skewed normal distribution

In this simulation, the sn package in R was used to generate skewed normal data. The skewed normal distribution is characterized by three parameters: location (ξ), scale (ω), and shape (α). The variance of a skewed normal distribution is $\omega^{2}\left(1-\frac{2\alpha^{2}}{\left(1+\alpha^{2}\right)\pi }\right)$ [38]. Two data sets with skewed normal distribution, with all the parameters being the same except for ω were created. To compare performances of these tests under different levels of skewness, two different values of α, 10, and 50 were separately simulated.

**Fig. 4:**
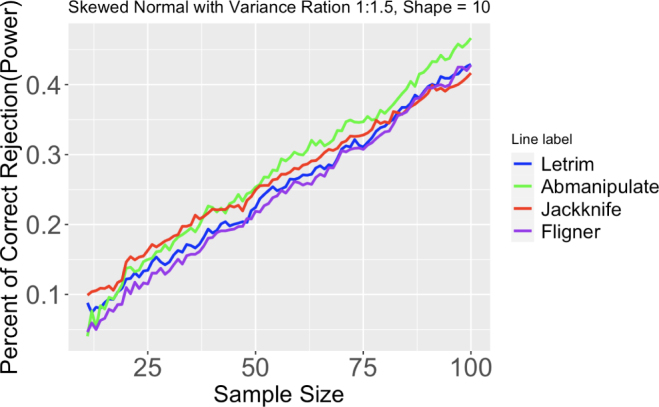
Proportion of correct rejection, or power, of different tests out of 2000 runs. Two skewed normal distributions have same means, but differ in variance ratio (1:1.5). Sample size ranges from 11 to 100. Shape = 10.

Please refer to Fig. 4, and figures from 17 to 23 for the following paragraph.

Given the large variance ratio and sample size, Levene’s test using trimmed mean has the highest rejection rates; when variance ratio and sample size decrease, the Jackknife method becomes the most powerful test. Levene’s test using the trimmed mean is constantly better than Levene’s test using the median. Ansari–Bradley test is almost unusable without adjusting the median for skewed normal data; despite the controversy of manually adjusting the median for the Ansari–Bradley test, this test performs well when the variance ratio is small. Note that as skewness increases, the rejection rates for all tests decrease for all variance ratios.

### 4.2 Three-sample simulations

#### 4.2.1 Data with normal distribution

To test for equal variances among 3 groups of normally distributed data, there are at least eight available tests to use: Levene’s test using the sample mean, Levene’s test using sample median, Levene’s test using trimmed mean, non-parametric Levene’s test, Bartlett’s test, Fligner–Killeen test, Hartley’s test, and Cochran’s test. The data are generated in such a way that the variances for two of the three groups are equally small while the third group has a larger variance. Using their rejection rates, we can assess these tests’ ability to identify unequal variances among the groups.

**Fig. 5:**
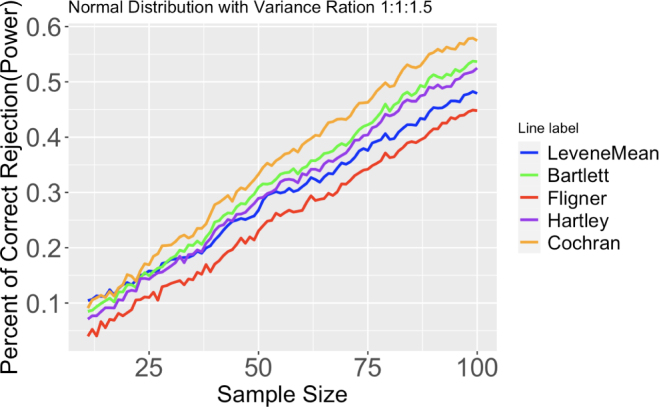
Proportion of correct rejection, or power, of different tests out of 2000 runs. Three normal distributions have same means, but differ in variance ratio (1:1:1.5). Sample size ranges from 11 to 100.

Please refer to Fig. 5, Fig. 24, Fig. 25, and Fig. 26 for the following paragraph.

In most cases, the results show that Cochran’s test is the most powerful test to detect differences in the variances, followed by Bartlett’s test and Hartley’s test. As the variance ratio and the sample size decrease, Hartley’s test starts to lose its advantage, and Levene’s test using sample means becomes preferable. Cochran’s test and Levene’s test using sample means have the highest rejection rate when the sample size is small.

#### 4.2.2 Data with heavy-tailed distribution

In this simulation, the group with the largest variance has a t-distribution of 3 degrees of freedom, and the other two groups are t-distributed with degrees of freedom of 12, 6, or 4. Hartley’s test and Cochran’s test cannot be used here due to the non-normality of the data.

**Fig. 6:**
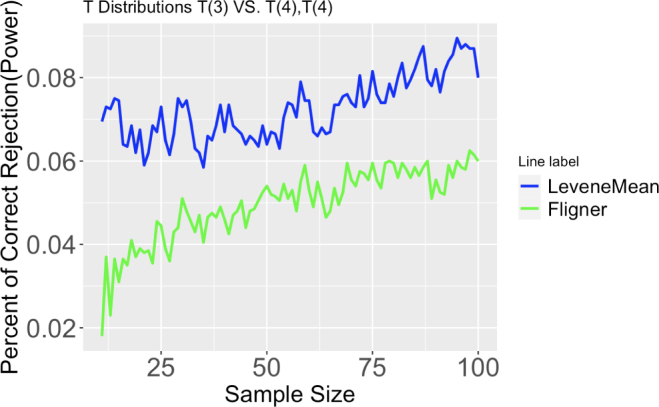
Proportion of correct rejection, or power, of different tests out of 2000 runs. Three T distributions with degrees of freedom 3, 4, and 4. Sample size ranges from 11 to 100.

Figures 6, 27, and 28 provide support for the findings describe in the following paragraph.

Levene’s test using sample mean is always the best test to use. With concerns of assumption violation or test validity, one can use Levene’s test using trimmed mean to get rid of heavy tails. Simulation results also show that the new non-parametric Levene’s test works well in detecting small variance differences in heavy-tailed data.

#### 4.2.3 Data with skewed normal distribution

In this simulation, I use the sn package in R to generate skewed normal data and test different combinations of ω and α. Two groups have the same skewed normal distribution, and the third group has the same parameters except for larger ω. In this scenario only 4 tests are available to use: Levene’s test using sample median, Levene’s test using trimmed mean, nonparametric Levene’s test, and Fligner–Killeen test.

Fig. 7 and those from Figs 29 to 35 provide support that Levene’s test using trimmed mean is always the most effective test to use. Fligner–Killeen test, although not as effective as Levene’s test using trimmed mean, performs fairly well in detecting small variance ratios.

**Fig. 7:**
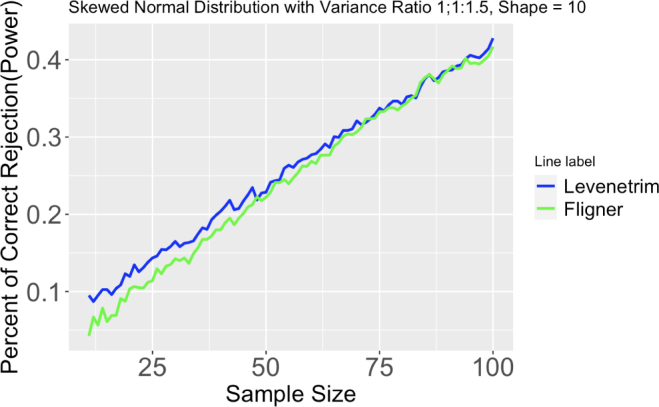
Proportion of correct rejection, or power, of different tests out of 2000 runs. Two skewed normal distributions have same means, but differ in variance ratio (1:1:1.5). Sample size ranges from 11 to 100.

### 4.3 Examining type one errors in variance homogeneity tests

**Fig. 8:**
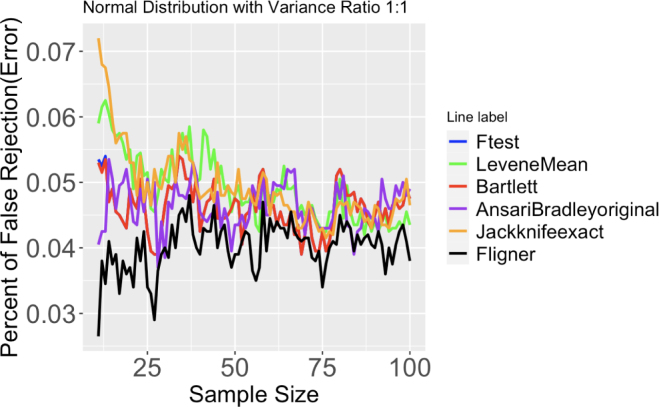
Proportion of false rejection, or error, of different tests out of 2000 runs. Two normal distributions with variance ratio 1:1. Sample size ranges from 11 to 100.

This subsection briefly examines how type 1 error rates are affected by variance heterogeneity when variance homogeneity tests were assumed. From results in our simulation, we graph the percentage of false rejection versus sample size in Fig. 8, and in Figs. 36 to 39, we obtain the following conclusions.

For a two-sample comparison in normal distributions, all tests have error rates below .05 when sample size is large. When the sample size is small, the error rates Levene’s test and Jackknife test are enlarged compared to other tests. For skewed distributions, most of the tests have error rates around .06 at high sample sizes. Bartlett’s test have error rates of almost .1 across most sample sizes, which are larger than any other tests at any sample sizes. In T distributions, available tests all have similar error rates around .05 at most sample sizes, except Jackknife test, whose error rates fluctuate around .08.

For a three-sample comparison in normal distributions, all tests have error rates below .05 when sample size is large. When sample size is small, the error rates of Levene’s tests are enlarged compared to other tests. In skewed distributions, Levene’s test using median has smaller error rates across other tests for any sample sizes. This conclusion is similar for t distributions.

### 4.4 Summary of simulation results

**Fig. 9:**
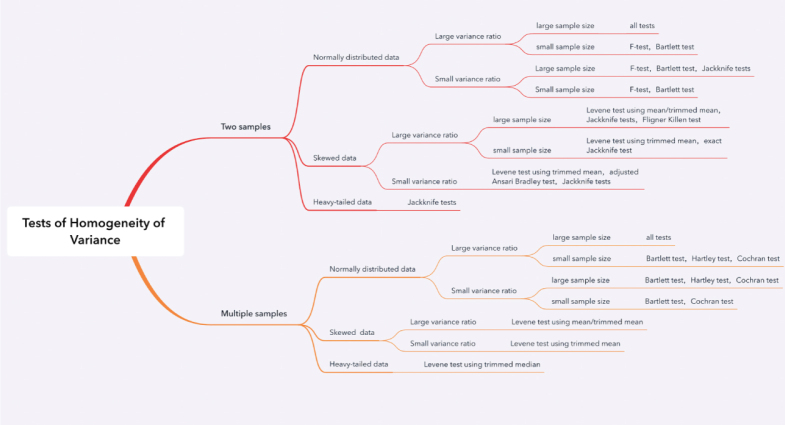
Our suggested roadmap when to use which tests for homogeneity of variance.

In this subsection, we use our simulation results and offer some guidance on what tests to use for testing variance homogeneity in during situations in clinical trials. The figure below succinctly displays a suggested roadmap to arrive at an appropriate test depending on the number of treatment groups involved, the anticipated type of distribution of the data, the variance ratios and sample sizes. One should read Fig. 9 from left to right across the various scenarios and arrive at a recommended test.

Generally, the Jackknife test is one test that one should use in two sample comparisons in terms of power. However, when considering error rates, Jackknife would not be a good choice for data with low sample sizes and heavy tails. For multi-sample comparisons, Levene’s test is a better choice for skewed normal data and heavy-tailed data compared to others. When multiple groups of data are normally distributed, the Bartlett test and Cochran test become great choices.

## 5 Shiny app

In this section, we describe the Shiny app and the R code that we have created to generate some of the simulation results. Readers may modify and run the code to replicate some results in the paper or generate new simulation results.

**Fig. 10:**
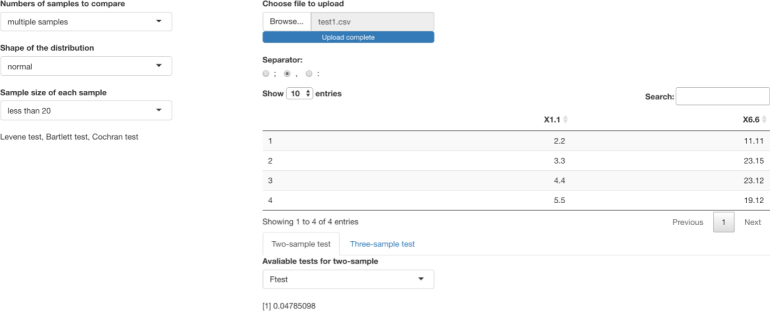
Shiny app page of choosing appropriate tests for homogeneity of variance and applying those tests to someone’s own data sets [39]. The number presented on the bottom right is the p-value of a specific test result of homogeneity of variance.

The figure below is a sample page from the app that helps readers select and perform a test of homogeneity (see Fig. 10).

The left column on this shiny page shows which tests are great to use in different sample sizes, different distributions, and different numbers of samples to compare. On the right side, one can upload a data set, and apply a specific test of homogeneity of variance from the left column and observe whether the test returns a significant result. Further details of the app are available at https://github.com/Jooooooeeee/Test-of-Homogeneity-of-Variance. For direct use of this app, please visit https://yuhangzhou533.shinyapps.io/Variance-Homogeneity/?_ga=2.78666452.1311773059.1677443373-585585766.1677443373.

We now illustrate how to analyze a subset of the real data from a two-arm clinical trial where Scleroderma patients were randomized to receive a high dose of D-Penicillamine (group = 1) or a low dose of D-Penicillamine (group = 0). The protocol required that patients showed up every 6 months for 2 years after the first baseline visit, resulting in a total of 4 visits for the duration of the study after the baseline visit. The main outcome is the skintot (skin thickness); the lower the score the less the patient is disabled by the disease. The main research question in the study was whether high dose of D-pen improves the skintot scores more significantly than a low dose of D- Penicillamine at 12th month. Details of the trial are available in Clements, el at. [40].

**Fig. 11:**
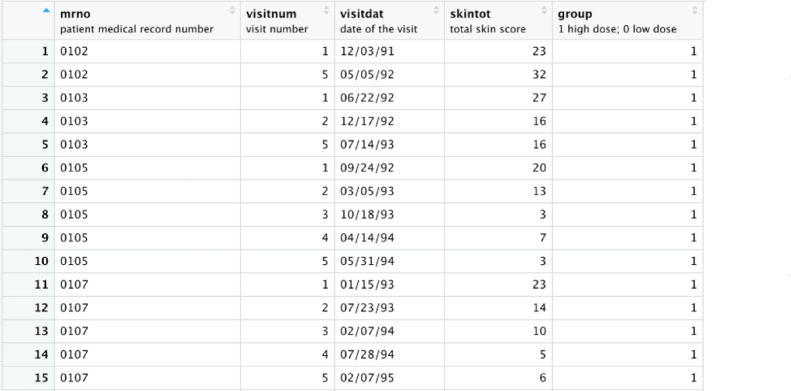
First 15 rows of a small subset of the longitudinal data set from the Clements’ study [14]. Each patient has a unique medical record number with up to five visit dates with visit 1 as the baseline visit. The skintot score is the main outcome of interest and clearly patients have missed visits. The table shows four patients who were randomized to the group with a high dose of D-pen (Group = 1).

Conventionally, to test for a treatment effect of a drug, a t-test may be used to compare the average skin total scores between the two groups at each visit, assuming responses from the two groups haves equal variance. More frequently, the change scores between the two groups are compared the end of the trial. For example, if the Jackknife test was employed to test homogeneity of variance at each time point, the app, after appropriate input, provides p-values of 0.27, 0.20, 0.28, 0.03, 0.28 respectively at each of the five time points. In this case, when using the t-test in R, it should be specified with equal variance = “FALSE” for the 4th visit (see Fig. 11).

## 6 Summary

There is a huge literature on different ways to assess whether there is heterogeneity in the data and this paper selectively focused on some homogeneity tests the are relevant to clinical trials. There are other useful distribution-free methods, including Lepage’s Rank test to test for equal location and dispersion of two samples simultaneously and Kolmogorov–Smirnov’s test to compare distributions of two groups. Lepage’s rank test combines Wilcoxon’s test and Ansari–Bradley’s statistic, and this new test is well-known for its consistency between exact and asymptotic estimates [41]. Kolmogorov–Smirnov’s test for distributions was developed based on smoothed distribution functions [42], [43]. Recent research has improved this test based on the Plachky–Steinebach theorem [43]. Researchers may consult related literature should they need to perform these tests for their study.

We note that there are other papers that compare relative merits of different methods for testing homogeneity of variance. For example, Rousson conducted an analysis of different variance homogeneity tests, its focus was on two-sample comparisons [44] and other papers tend to emphasize on normal data. This review aims to provide general advice on use of various methods to assess heterogeneity in clinical data using simulation-based results.

In the 2-sample scenarios, the Jackknife test, as a non-parametric method, works surprisingly well even when the data is normally distributed. When the sample size is large enough and the distribution is normal, we recommend the F-test or Bartlett’s test. When the distribution is skewed normal or heavily tailed, the sample size is too small to ascertain the normality assumption, or the goal is to detect a small difference in variances, the Jackknife test seems to be a good tool.

In multi-sample scenarios, Cochran’s C test is preferred for normally distributed data. For heavy-tailed or skewed normal data, some adjusted variants of Levene’s test (using trimmed mean or median) are preferable, which partially coincides with David and Brunos’ result for stating a preference for median [45]. Fligner–Killeen test may be preferable when one wishes to detect a small difference among the group variances of skewed normal data.

In conclusion, we have reviewed tests of homogeneity of variances of responses from patients across treated groups in clinical trials and used a simulation to investigate the robustness of the various tests to the homoscedasticity assumption. Based on our simulation results, we found that among the tests compared in the paper, there are some that seem preferable to others. For two-sample problems, the Jackknife method tends to outperform others regardless of the variance ratio or the sample size. For more than two groups, Barlett’s test and Cochran’s test are better choices when data are nearly normally distributed; otherwise, Levene’s test appears to be a better choice for non-normally distributed data.

We also observe that when the sample size is small, all the tests generally do not perform well. When data appear heteroscedastic, [21] proposed an alternative method to the F-test for testing variance homogeneity, and [46] proposed a data analytical strategy to preserve the type 1 error rate.

When data are heavily skewed, although there are better test options available based on the findings of this review, there appears to be no single test that performs well overall. In particular, most rank-based tests do not have good power and error rate performance, and further studies should be conducted to find more powerful tests for data that are heavily skewed [45].

When it is anticipated that heterogeneous responses vary systematically in a certain pattern, optimal design strategies can be used to provide best estimates for the model parameters at minimal cost. For example, Wong and Zhu [46] assumed variances of responses from different treatment groups vary predictably and found an optimal allocation scheme for subjects in the trial [46]. These designs depend on the unknown variance from the different groups and they can be implemented once nominal values for them are available, either from previous studies or from similar trials. More recently, Mavrogonato compared allocation strategies for optimizing clinical trial designs under various heteroscedastic assumptions [30], [47].

A limitation of the current paper is that it does not discuss use of adaptive designs to check model assumptions periodically during the trial and use accumulating data to amend the study design for more effective inference. There is a huge literature on adaptive designs with many and continuing enhancements in various ways to design and analyze clinical trial data, including how to check for variance homogeneity as data come in. However, space precludes us from covering this important topic adequately and fairly, and so we defer a fuller discussion of adaptive strategies to the near future.

## Data Availability

Data will be made available on request

## Appendix A Supplementary data

MMC S1 The supplementary material contains all simulations results for the percent of correct rejection in two/three data samples comparison, for all different variance ratios discussed during the paper, under all distribution assumptions discussed in the paper, with sample sizes ranging from 11 to 100.

## References

[1] Knief U., Forstmeier W. (2021). Violating the normality assumption may be the lesser of two evils. Behav. Res. Methods.

[2] Liang J., Tang M.-L., Zhao X. (2018). Testing high-dimensional normality based on classical skewness and kurtosis with a possible small sample size. Comm. Statist. Theory Methods.

[3] Orcanm F. (2020). Parametric or non-parametric: Skewness to test normality for mean comparison. Int. J. Assess. Tools Educ..

[4] Rietveld T., van Hout R. (2015). The t test and beyond: Recommendations for testing the central tendencies of two independent samples in research on speech, language and hearing pathology. J. Commun. Disord..

[5] Cain M.K., Zhang Z., Yuan K. (2017). Univariate and multivariate skewness and kurtosis for measuring nonnormality: Prevalence, influence and estimation. Behav. Res..

[6] Netuveli G. (2022). Cluster randomized controlled trial: a matter of independence. Int. J. Qual. Health Care.

[7] Scariano S.M., Davenport J.M. (1987). The effects of violations of independence assumptions in the one-way ANOVA. Amer. Statist..

[8] Edgell S.E., Noon S.M. (1984). Effect of violation of normality on the t test of the correlation coefficient. Psychol. Bull..

[9] Kédagni D., Mourifié I. (2020). Generalized instrumental inequalities: Testing the instrumental variable independence assumption. Biometrika.

[10] Visser M., Depaoli S. (2022). A guide to detecting and modeling local dependence in latent class analysis models. Struct. Equ. Model.: Multidiscip. J..

[11] Stephanie (2021). https://www.statisticshowto.com/comparison-of-means/.

[12] Abbas Z., Nazir H.Z., Akhtar N., Abid M., Riaz M. (2022). Non-parametric progressive signed-rank control chart for monitoring the process location. J. Stat. Comput. Simul..

[13] Frostig T., Benjamini Y. (2021). Testing the equality of multivariate means when p>n by combining the hotelling and Simes tests. TEST.

[14] RStudio Team (2020). http://www.rstudio.com/.

[15] R Core Team (2022). https://www.R-project.org/.

[16] Signorell Andri (2023). https://CRAN.R-project.org/package=DescTools.

[17] Sweet D. Lukke (2020). https://CRAN.R-project.org/package=nonpar.

[18] Gastwirth Joseph L., Gel Yulia R., Hui W.L. Wallace, Lyubchich Vyacheslav, Miao Weiwen, Noguchi Kimihiro (2022). https://CRAN.R-project.org/package=lawstat.

[19] Sandrini-Neto Leonardo, Camargo Mauricio G. (2022).

[20] Moder K. (2007). How to keep the type I error rate in ANOVA if variances are heteroscedastic. Austrian J. Stat..

[21] Dudewicz E.J., Ahmed S.U. (1999). New exact and asymptotically optimal heteroscedastic statistical procedures and tables, II. Am. J. Math. Manag. Sci..

[22] Eftekhar S., Sadooghi-Alvandi M., Kharrati-Kopaei M. (2017). Testing the equality of several multivariate normal mean vectors under heteroscedasticity: A fiducial approach and an approximate test. Comm. Statist. Theory Methods.

[23] Ansari A.R., Bradley R.A. (1960). Rank-sum tests for dispersions. Ann. Math. Stat..

[24] Moses L.E. (1963). Rank tests of dispersion. Ann. Math. Stat..

[25] Miller R.G. (1968). Jackknifing variances. Ann. Math. Stat..

[26] Levene H., Olkin I., Hotelling H. (1960). Contributions to Probability and Statistics: Essays in Honor of Harold Hotelling.

[27] Snedecor G.W., Cochran W.G. (1989).

[28] StataCorp (2021).

[29] Hartley H.O. (1950). The use of range in analysis of variance. Biometrika.

[30] Frey J. (2010). Testing for equivalence of variances using Hartley’s ratio. Canad. J. Statist..

[31] Lam Rue t́ (2010). Scrutiny of variance results for outliers: Cochran’s test optimized. Anal. Chim. Acta.

[32] Welch B.L. (1951). On the comparison of several mean values: An alternative approach. Biometrika.

[33] Brown M.B., Forsythe A.B. (1974). Robust tests for the equality of variances. J. Amer. Statist. Assoc..

[34] Nordstokke D.W., Zumbo B.D. (2010). A new nonparametric levene test for equal variances. Psicológica.

[35] Conover W.J., Johnson M.E., Johnson M.M. (1981). A comparative study of tests for homogeneity of variances, with applications to the outer continental shelf bidding data. Technometrics.

[36] Azzalini A. (1985). A class of distributions which includes the normal ones. Scand. J. Stat..

[37] Kropf S. (2000). Multiple comparisons of treatments with stable multivariate tests in a two-stage adaptive design, including a test for non-inferiority. Biom. J..

[38] Lepage YVES (1971). A combination of Wilcoxon’s and Ansari-Bradley’s statistics. Biometrika.

[39] Chang Winston, Cheng Joe, Allaire JJ, Sievert Carson, Schloerke Barret, Xie Yihui, Allen Jeff, McPherson Jonathan, Dipert Alan, Borges Barbara (2022). https://CRAN.R-project.org/package=shiny.

[40] Clements P.J., Furst D.E., Wong W.-K., Mayes M., White B., Wigley F. (1999). High-dose versus low-dose D-penicillamine in early diffuse systemic sclerosis: Analysis of a two-year, double-blind, randomized, controlled clinical trial. Arthritis Rheum.

[41] Chakravarti, Laha, Roy (1967).

[42] Butorina Y.O., Nikitin Y.Y. (2011).

[43] Nordstokke D.W., Zumbo B.D. (2009). A new nonparametric levene test for equal variances. Psicologica: Int. J. Methodol. Exp. Psychol..

[44] Rousson V. (2002). On distribution-free tests for the multivariate two-sample location-scale model. J. Multivariate Anal..

[45] (2010). Heterogeneity of variances (a simulation study). Psychol. Test Assess. Model..

[46] Wong W.K., Zhu W. (2008). Optimal subject allocation scheme to various treatment groups under a variance heterogenity model. Stat. Med..

[47] Cochran W.G. (1941). The distribution of the largest of a set of estimated variances as a fraction of their total. Ann. Hum. Genet. (London).

