# Supplementary material for: Statistical tests for homogeneity of variance for clinical trials and recommendations
Source: Contemp Clin Trials Commun. 2023 Mar 31;33:101119. doi: 10.1016/j.conctc.2023.101119 (PMC10151260; doi:10.1016/j.conctc.2023.101119)
Supplement: MMC S1 — The supplementary material contains all simulations results for the percent of correct rejection in two/three data samples comparison, for all different variance ratios discussed during the paper, under all distribution assumptions discussed in the paper, with sample sizes ranging from 11 to 100. [file mmc1.pdf]

Supplemental materials

The supplement materials contain various plots that support our simulations results discussed in the paper.

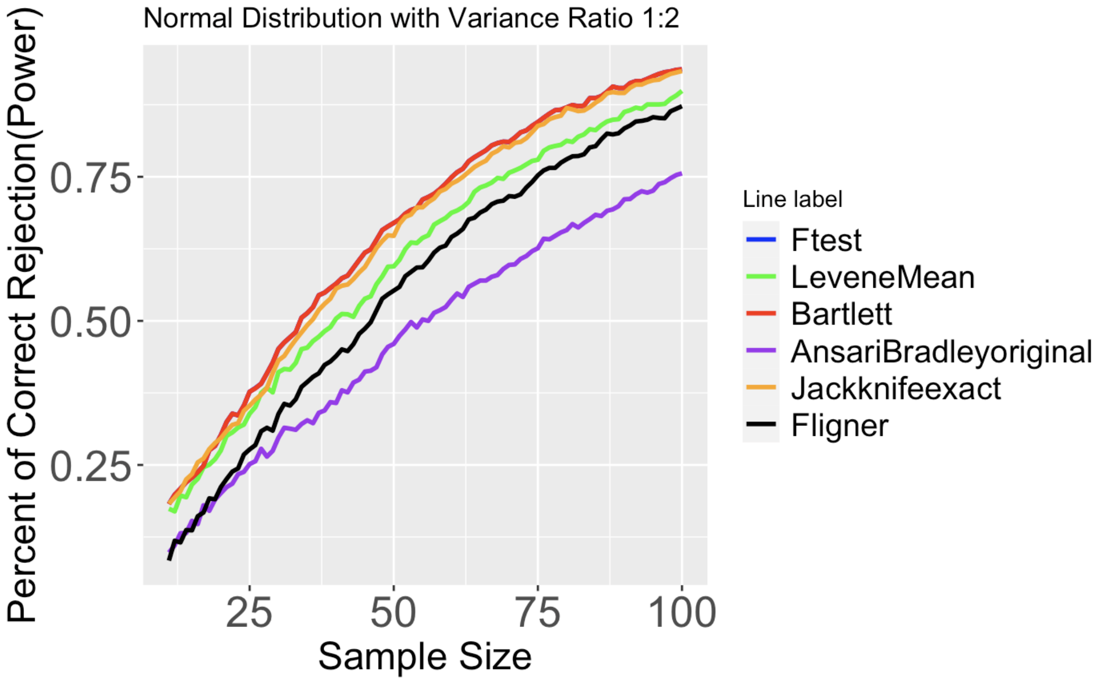

Figure 12: Proportion of correct rejection, or power, of different tests out of 2000 runs. Two normal distributions have same means, but differ in variance ratio(1:2). Sample size ranges from 11 to 100.

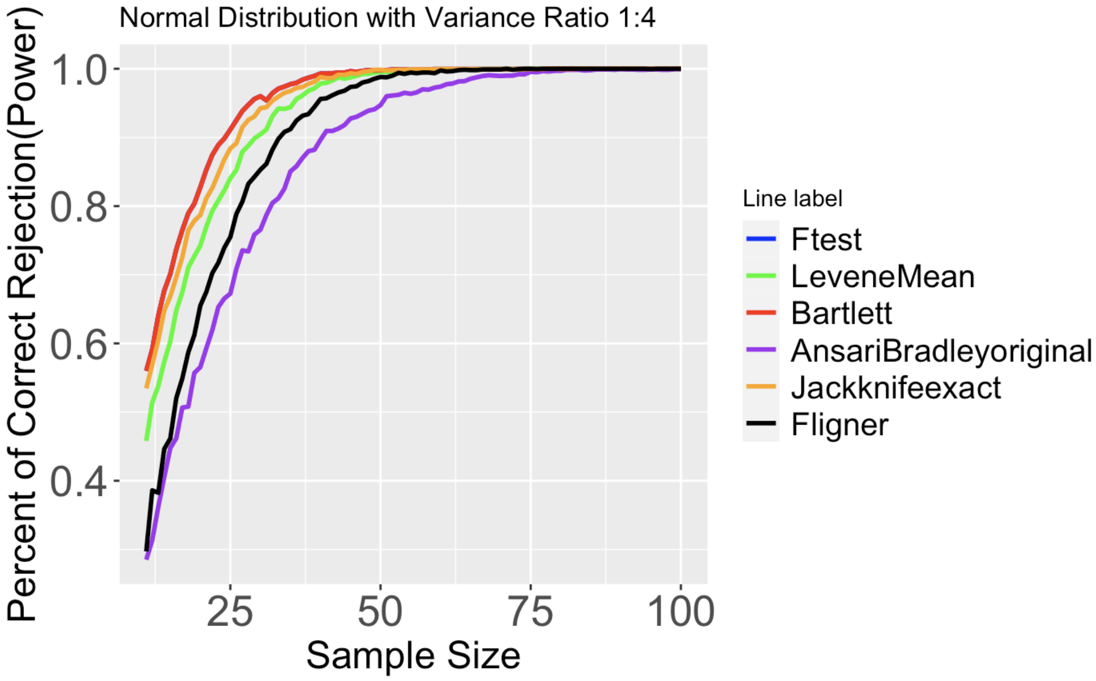

Figure 13: Proportion of correct rejection, or power, of different tests out of 2000 runs. Two normal distributions have same means, but differ in variance ratio(1:4). Sample size ranges from 11 to 100.

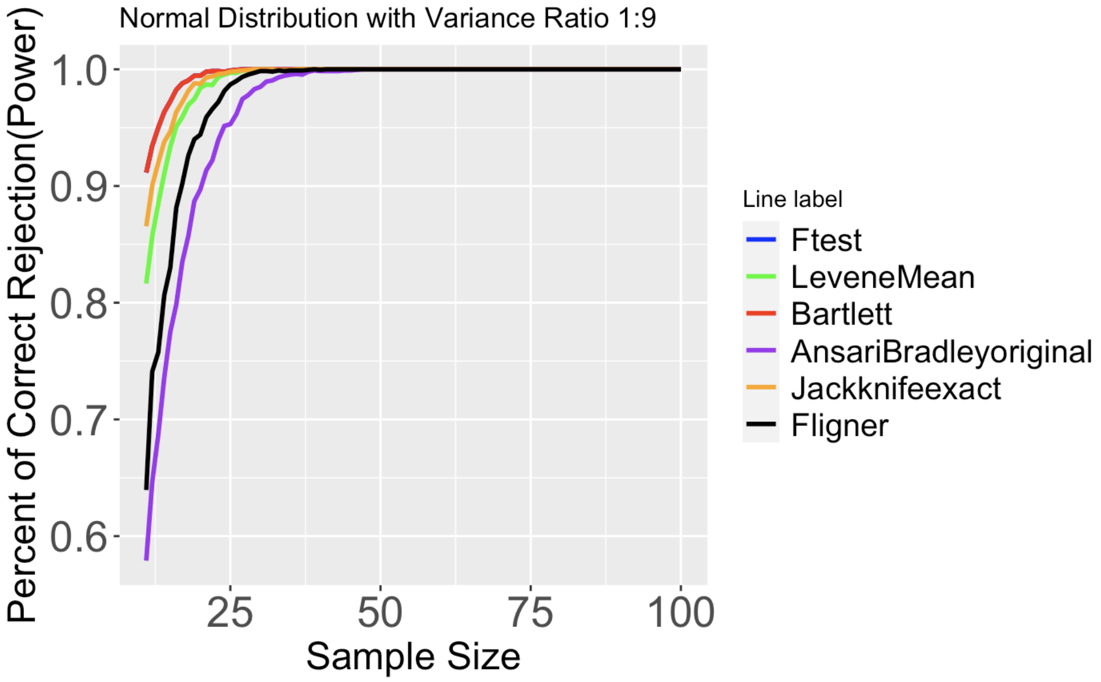

Figure 14: Proportion of correct rejection, or power, of different tests out of 2000 runs. Two normal distributions have same means, but differ in variance ratio(1:9).

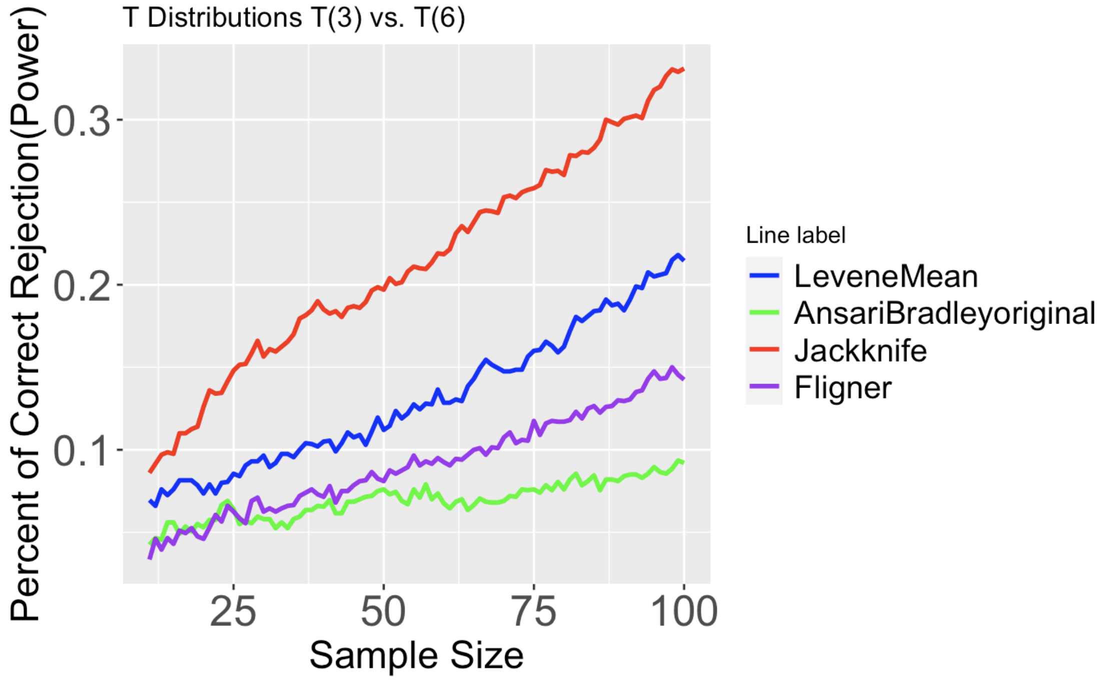

Figure 15: Proportion of correct rejection, or power, of different tests out of 2000 runs. Two T distributions have degree of freedoms 3 and 6. Sample size ranges from 11 to 100.

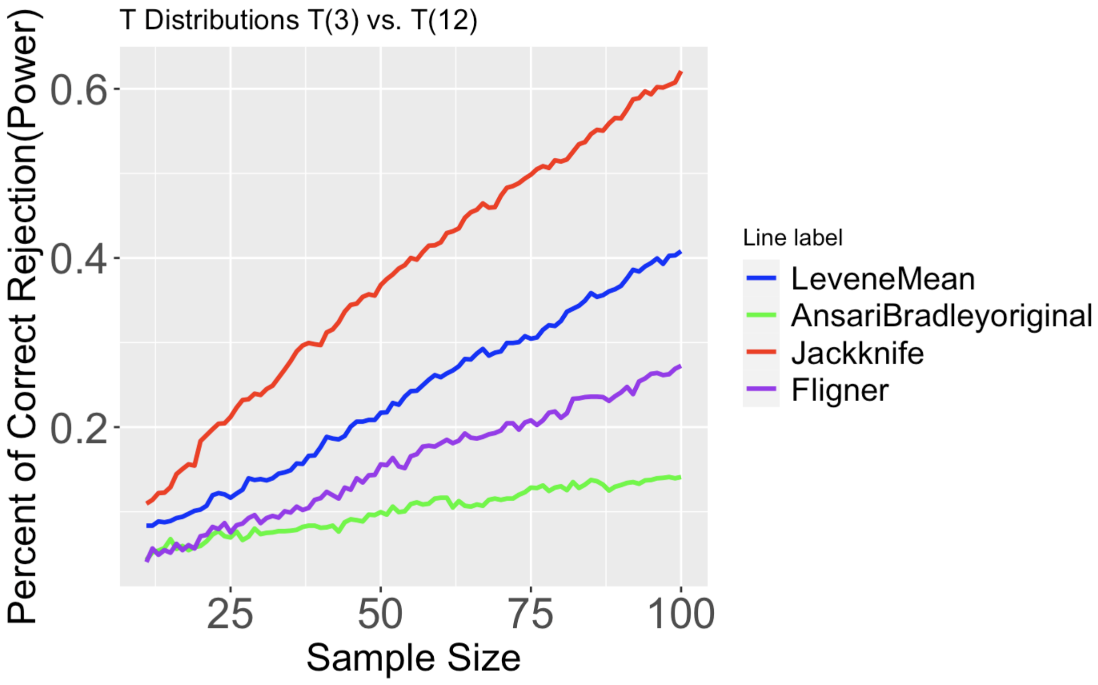

Figure 16: Proportion of correct rejection, or power, of different tests out of 2000 runs. Two T distributions have degree of freedoms 3 and 12. Sample size ranges from 11 to 100.

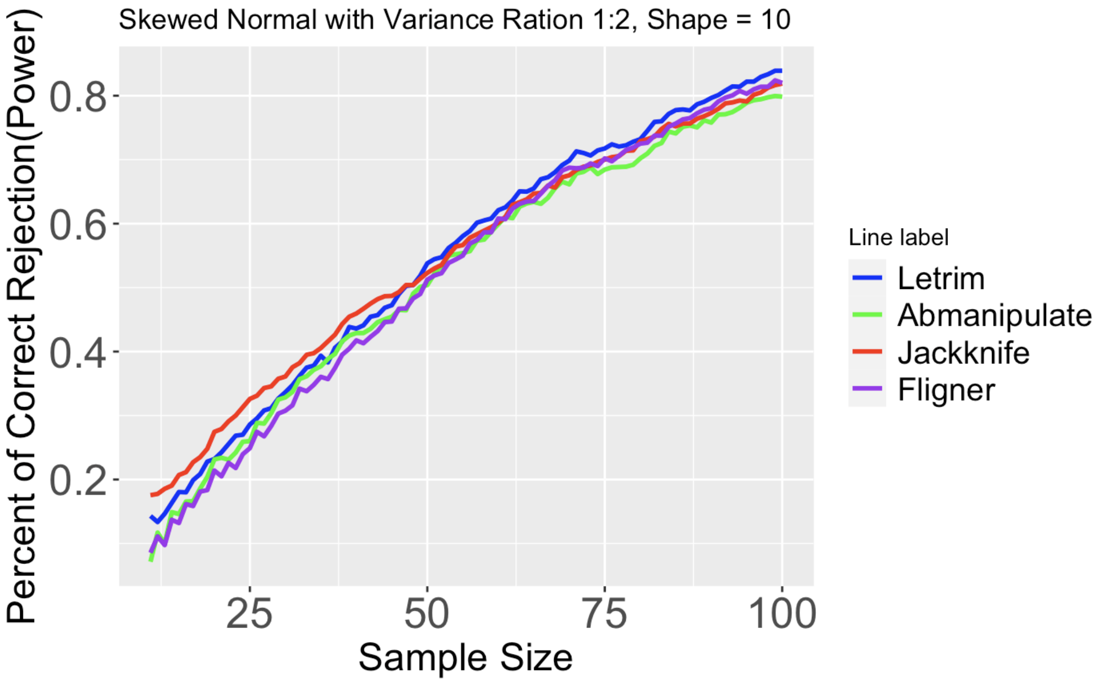

Figure 17: Proportion of correct rejection, or power, of different tests out of 2000 runs. Two skewed normal distributions(shape = 10) have same mean, but differ in variance ratio(1:2). Sample size ranges from 11 to 100.

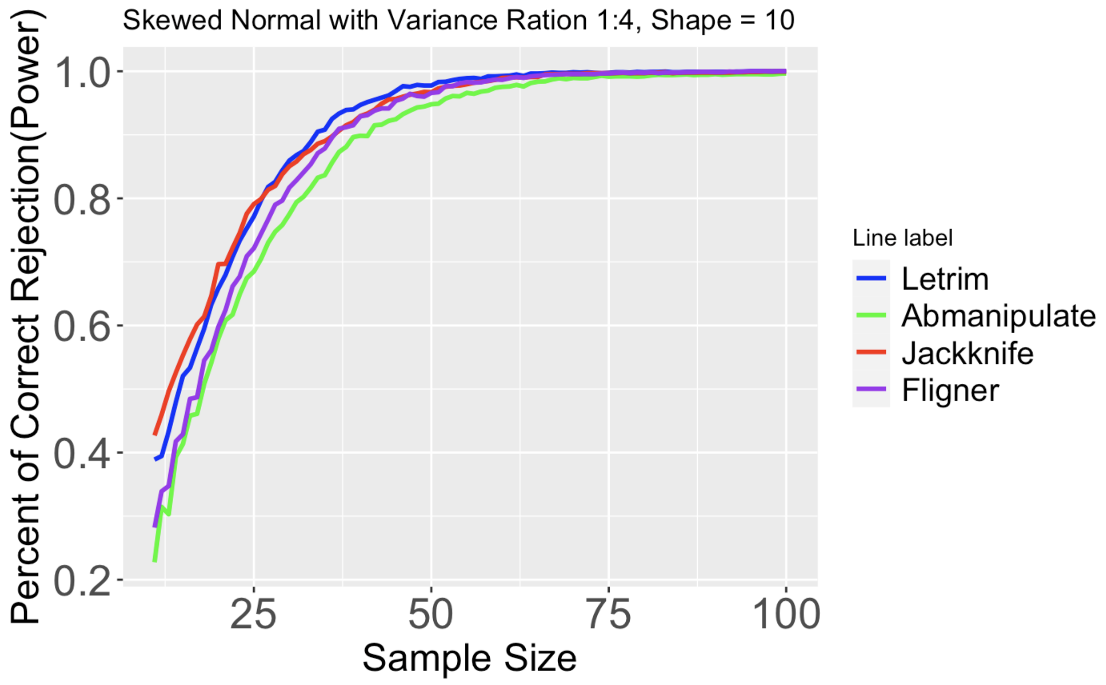

Figure 18: Proportion of correct rejection, or power, of different tests out of 2000 runs. Two skewed normal distributions(shape = 10) have same mean, but differ in variance ratio(1:4). Sample size ranges from 11 to 100.

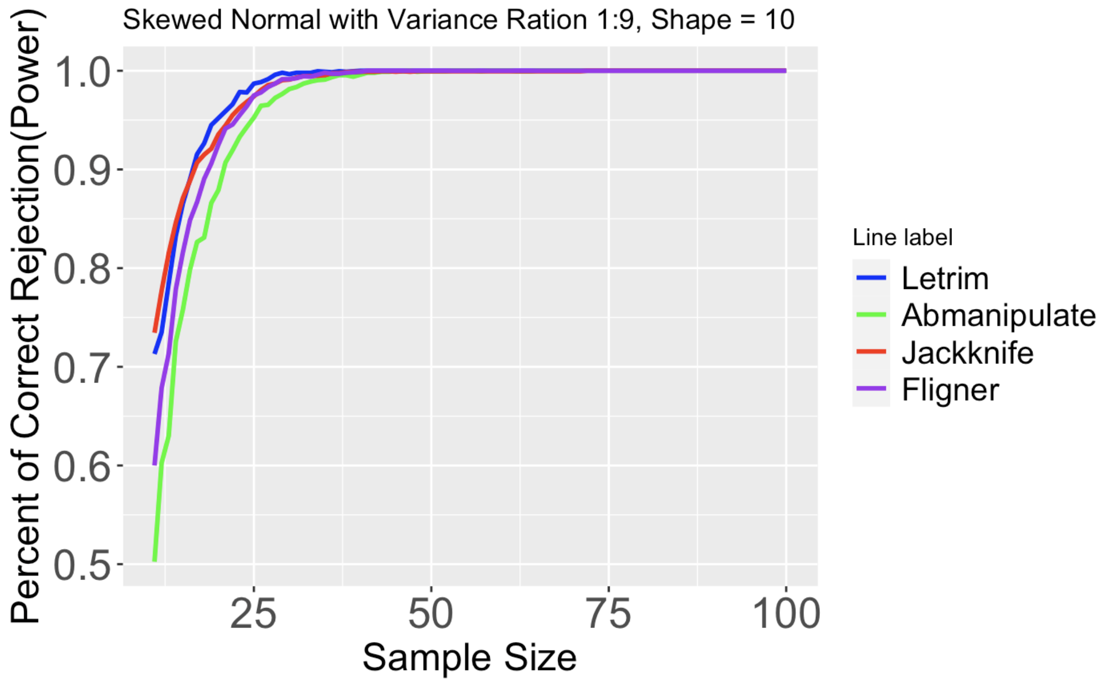

Figure 19: Proportion of correct rejection, or power, of different tests out of 2000 runs. Two skewed normal distributions(shape = 10) have same mean, but differ in variance ratio(1:9).

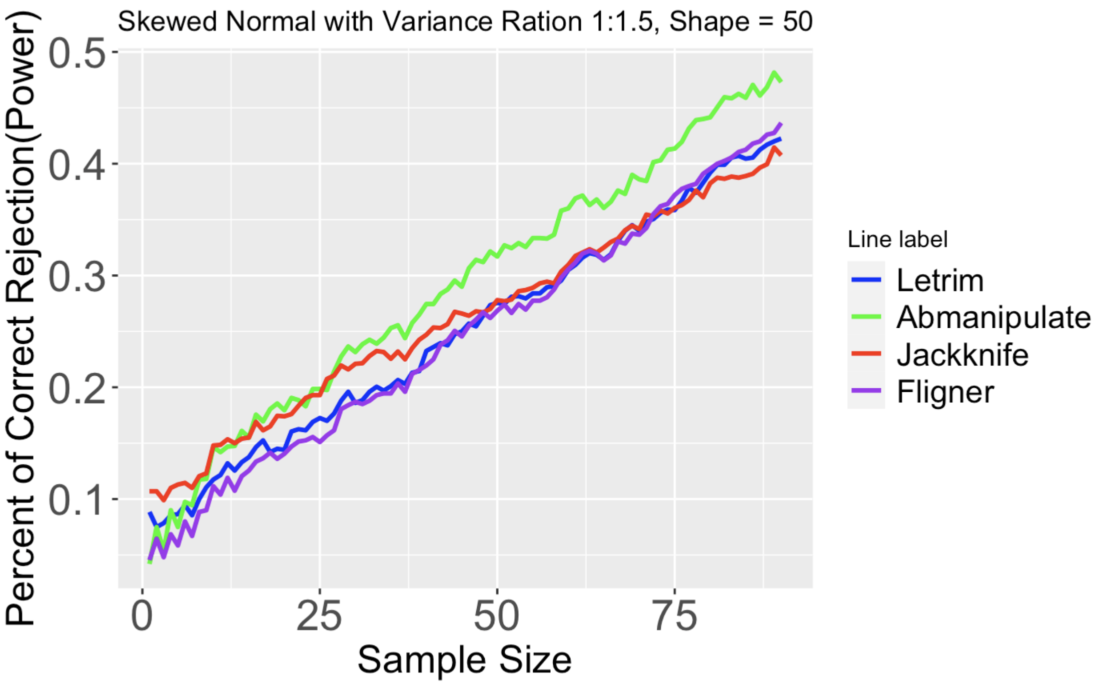

Figure 20: Proportion of correct rejection, or power, of different tests out of 2000 runs. Two skewed normal distributions(shape = 50) have same mean, but differ in variance ratio(1:1.5). Sample size ranges from 11 to 100.

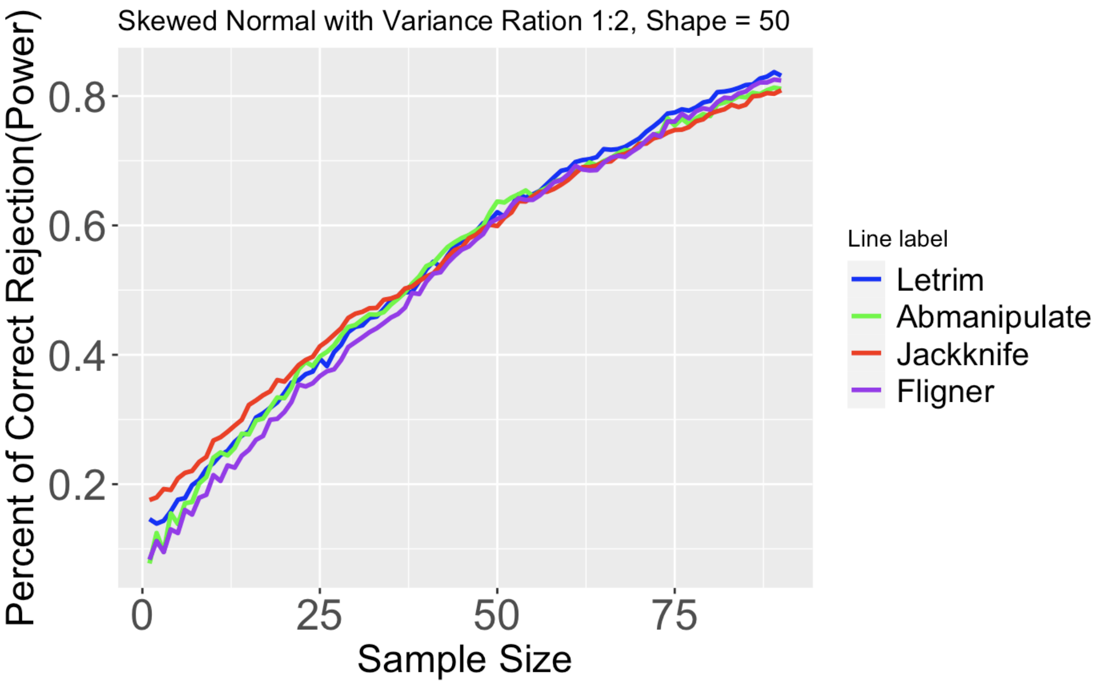

Figure 21: Proportion of correct rejection, or power, of different tests out of 2000 runs. Two skewed normal distributions(shape = 50) have same mean, but differ in variance ratio(1:2). Sample size ranges from 11 to 100.

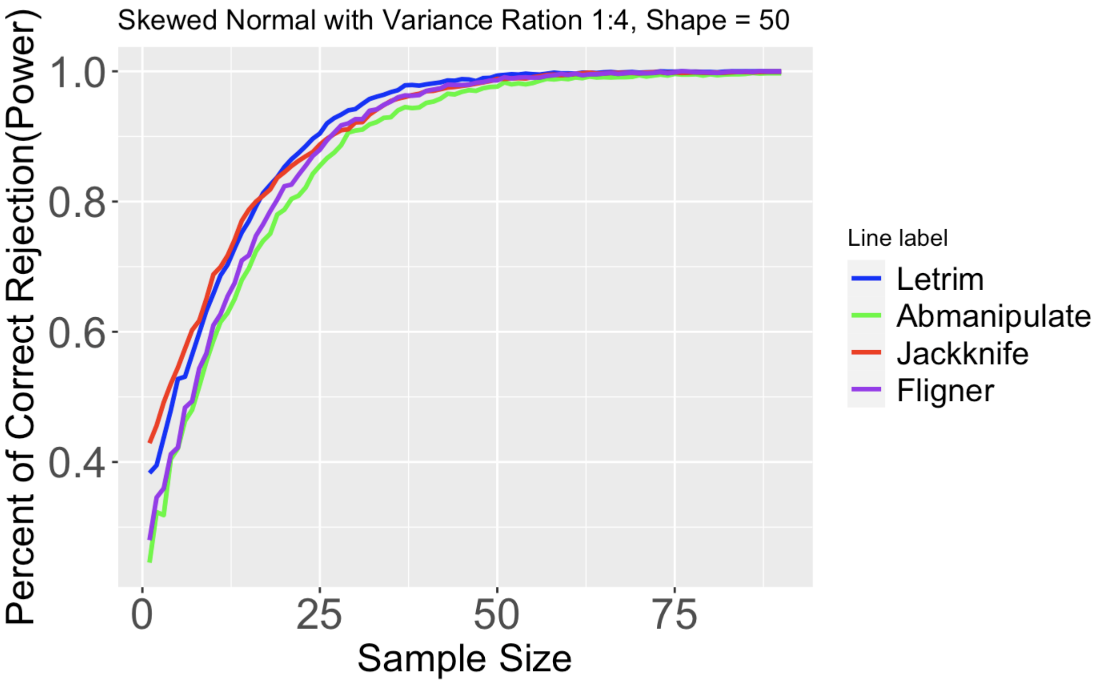

Figure 22: Proportion of correct rejection, or power, of different tests out of 2000 runs. Two skewed normal distributions(shape = 50) have same mean, but differ in variance ratio(1:4). Sample size ranges from 11 to 100.

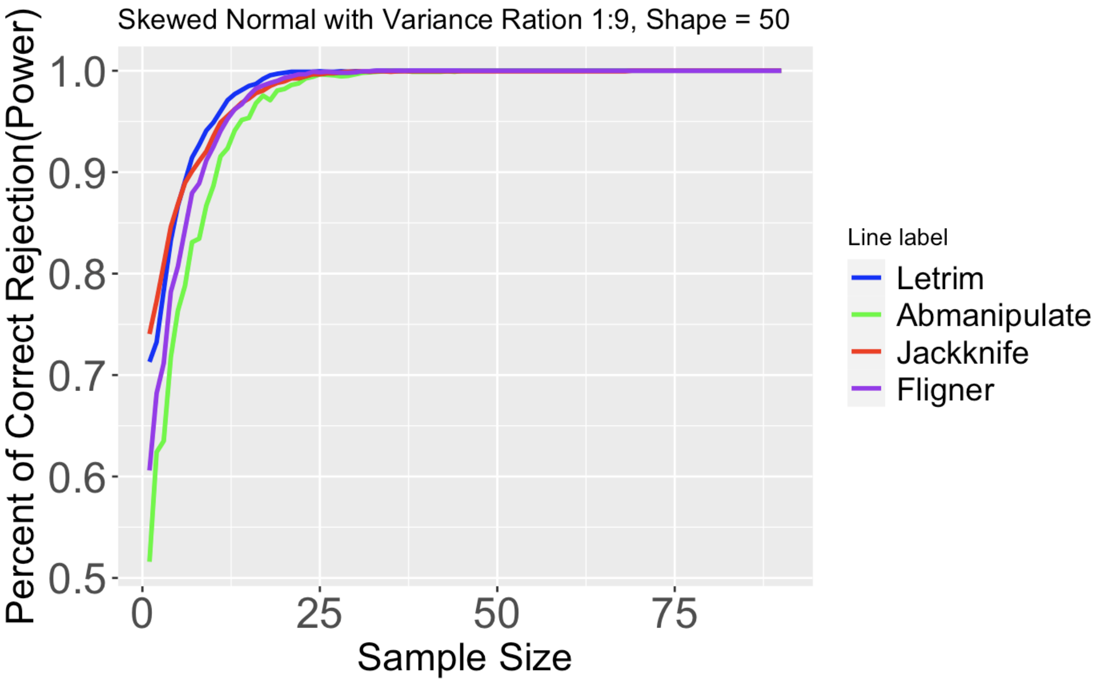

Figure 23: Proportion of correct rejection, or power, of different tests out of 2000 runs. Two skewed normal distributions(shape = 50) have same mean, but differ in variance ratio(1:9). Sample size ranges from 11 to 100.

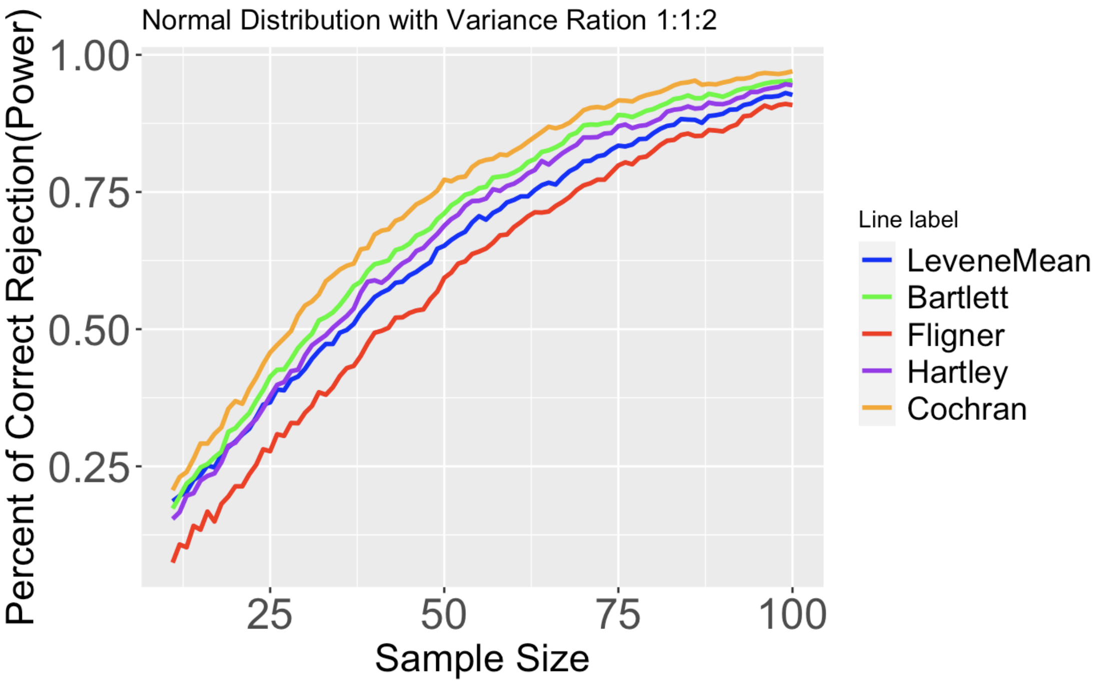

Figure 24: Proportion of correct rejection, or power, of different tests out of 2000 runs. Three normal distributions have same means, but differ in variance ratio(1:1:2). Sample size ranges from 11 to 100.

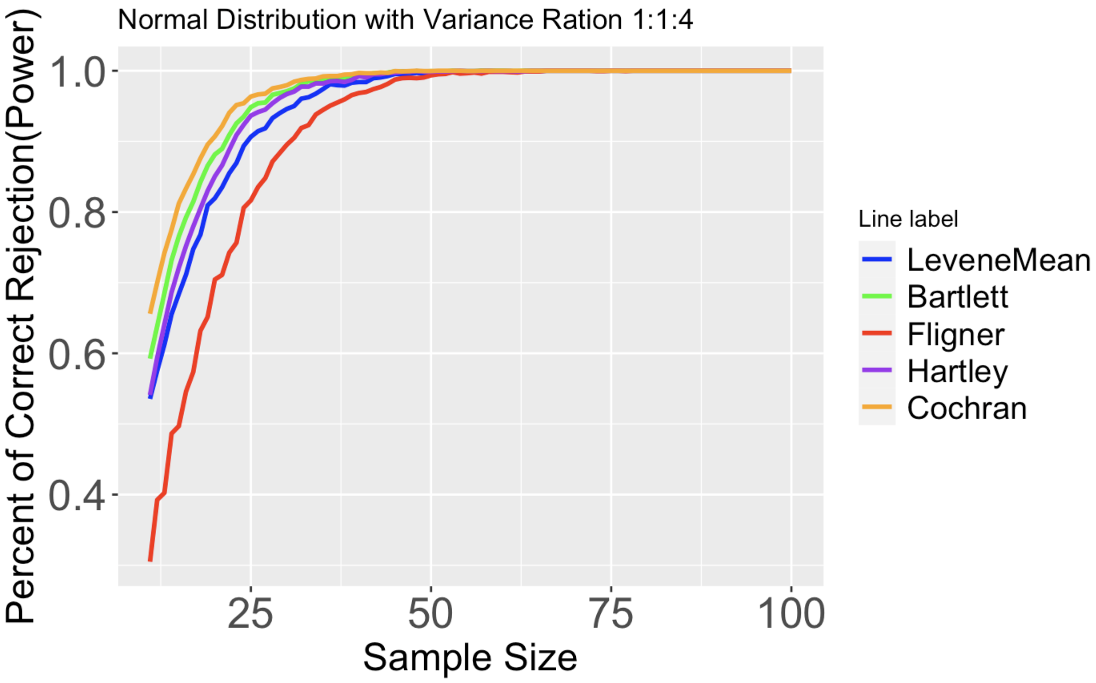

Figure 25: Proportion of correct rejection, or power, of different tests out of 2000 runs. Three normal distributions have same means, but differ in variance ratio(1:1:4). Sample size ranges from 11 to 100.

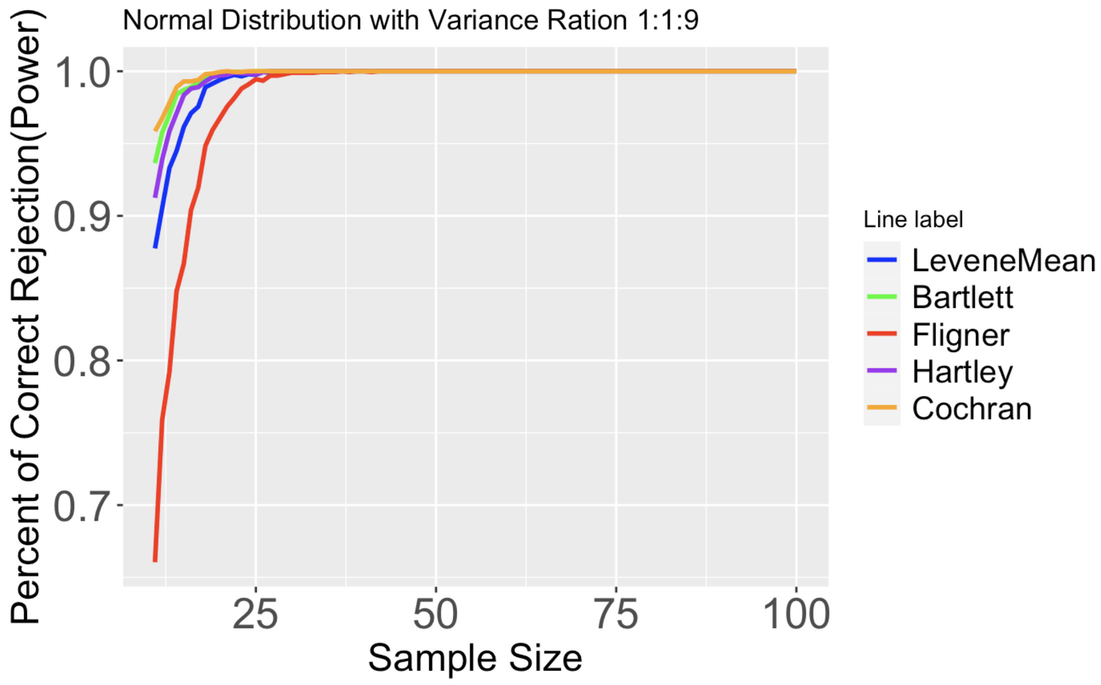

Figure 26: Proportion of correct rejection, or power, of different tests out of 2000 runs. Three normal distributions have same means, but differ in variance ratio(1:1:9). Sample size ranges from 11 to 100.

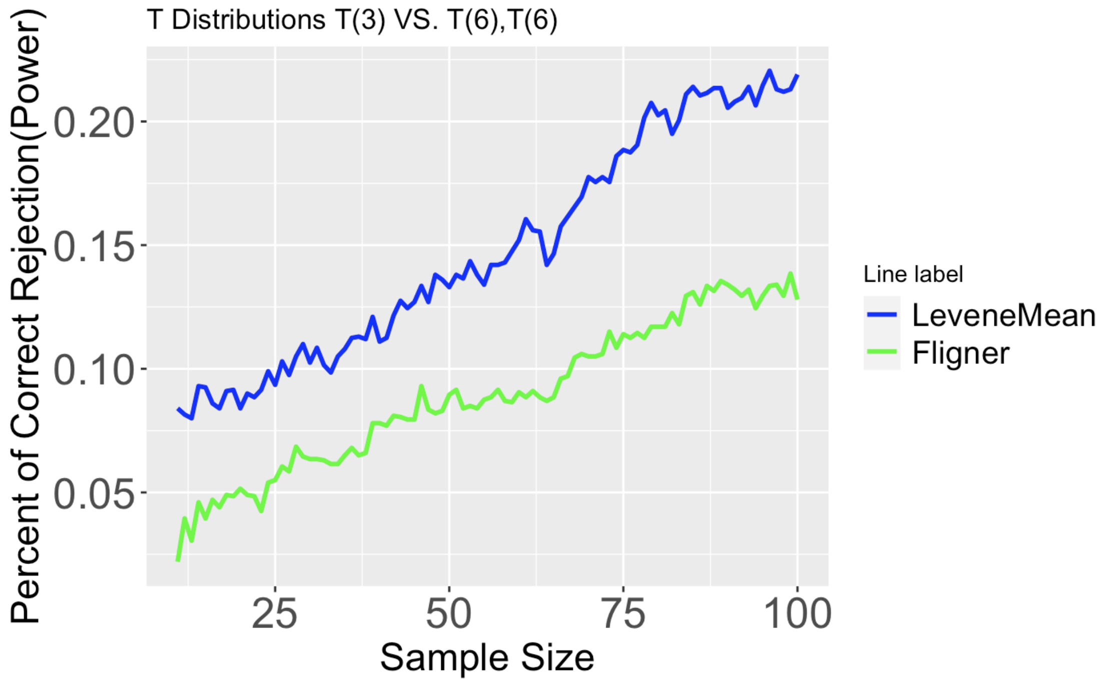

Figure 27: Proportion of correct rejection, or power, of different tests out of 2000 runs. three T distributions have degree of freedoms 3, 6, and 6. Sample size ranges from 11 to 100.

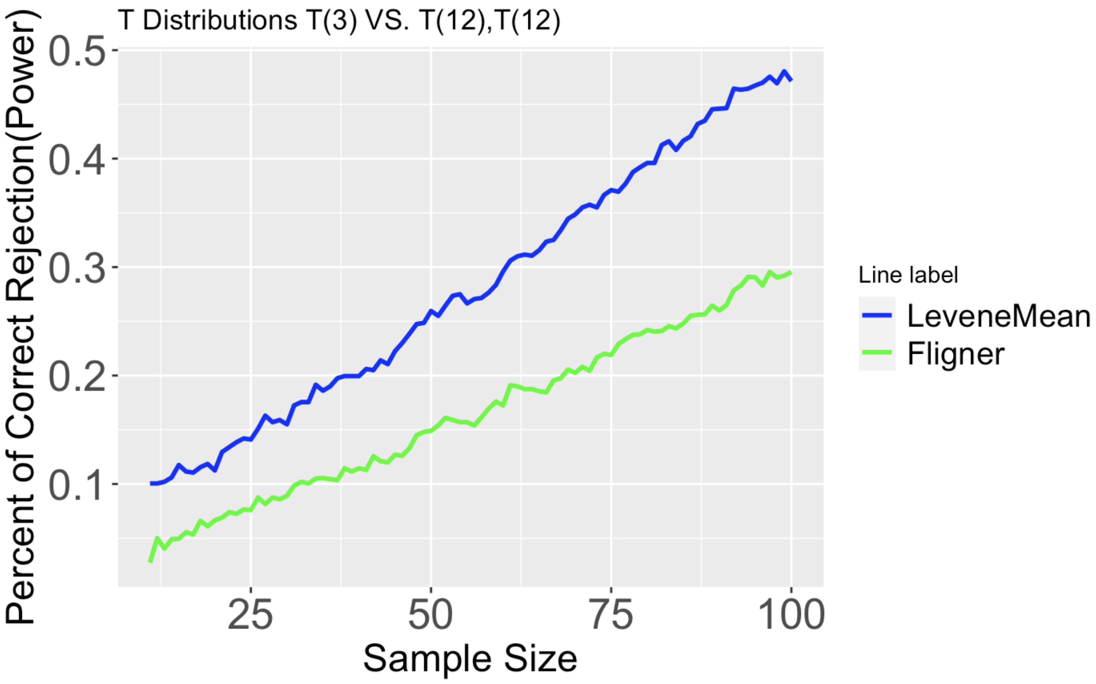

Figure 28: Proportion of correct rejection, or power, of different tests out of 2000 runs. three T distributions have degree of freedoms 3, 12, and 12. Sample size ranges from 11 to 100.

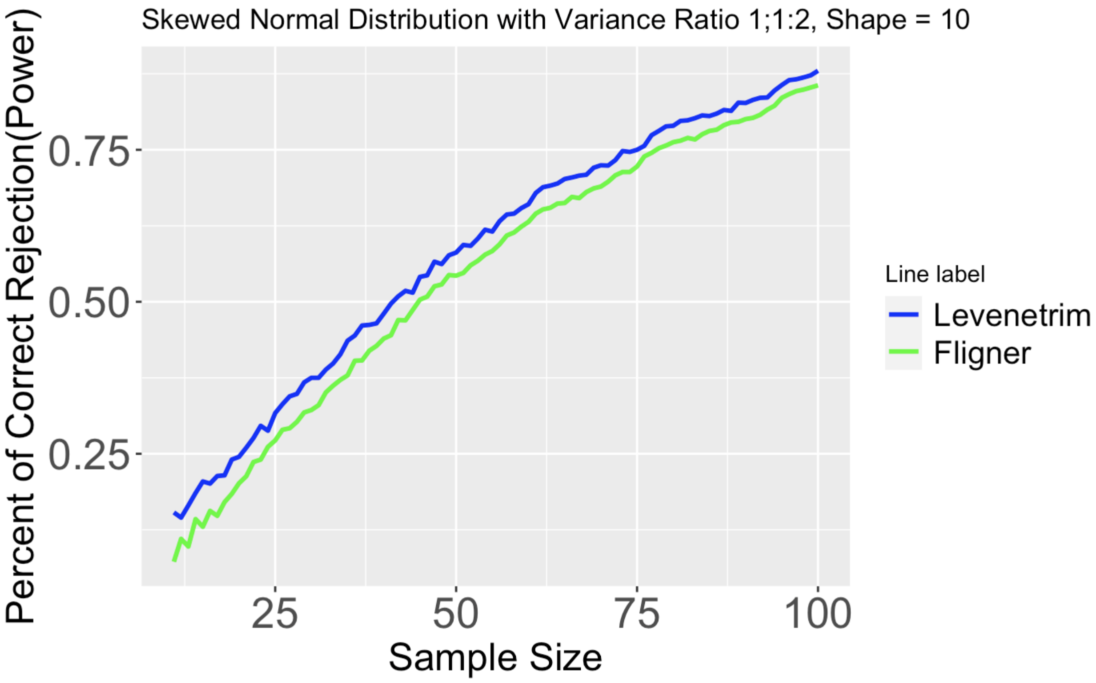

Figure 29: Proportion of correct rejection, or power, of different tests out of 2000 runs. Three skewed normal distributions(shape = 10) have same mean, but differ in variance ratio(1:1:2). Sample size ranges from 11 to 100.

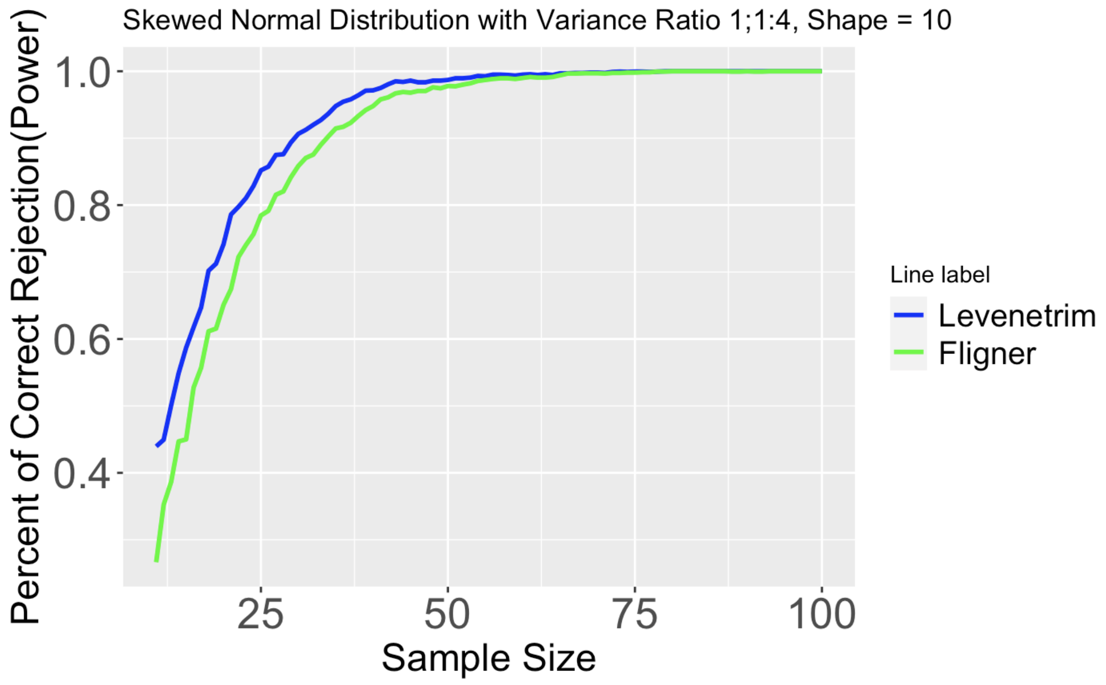

Figure 30: Proportion of correct rejection, or power, of different tests out of 2000 runs. Three skewed normal distributions(shape = 10) have same mean, but differ in variance ratio(1:1:4). Sample size ranges from 11 to 100.

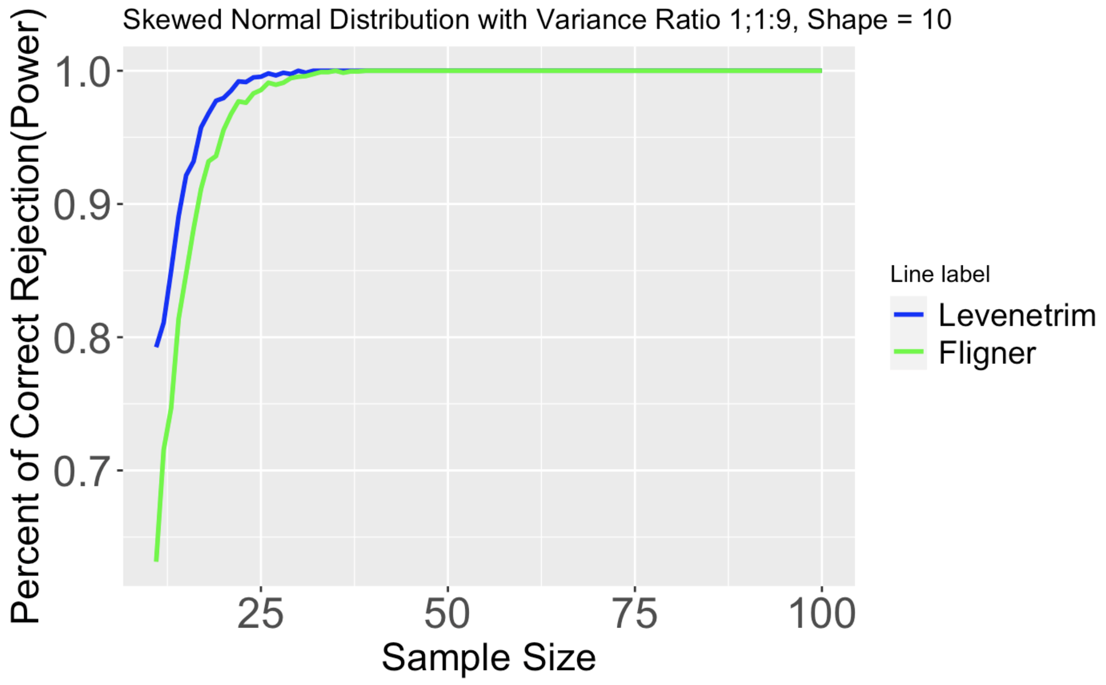

Figure 31: Proportion of correct rejection, or power, of different tests out of 2000 runs. Three skewed normal distributions(shape = 10) have same mean, but differ in variance ratio(1:1:9). Sample size ranges from 11 to 100.

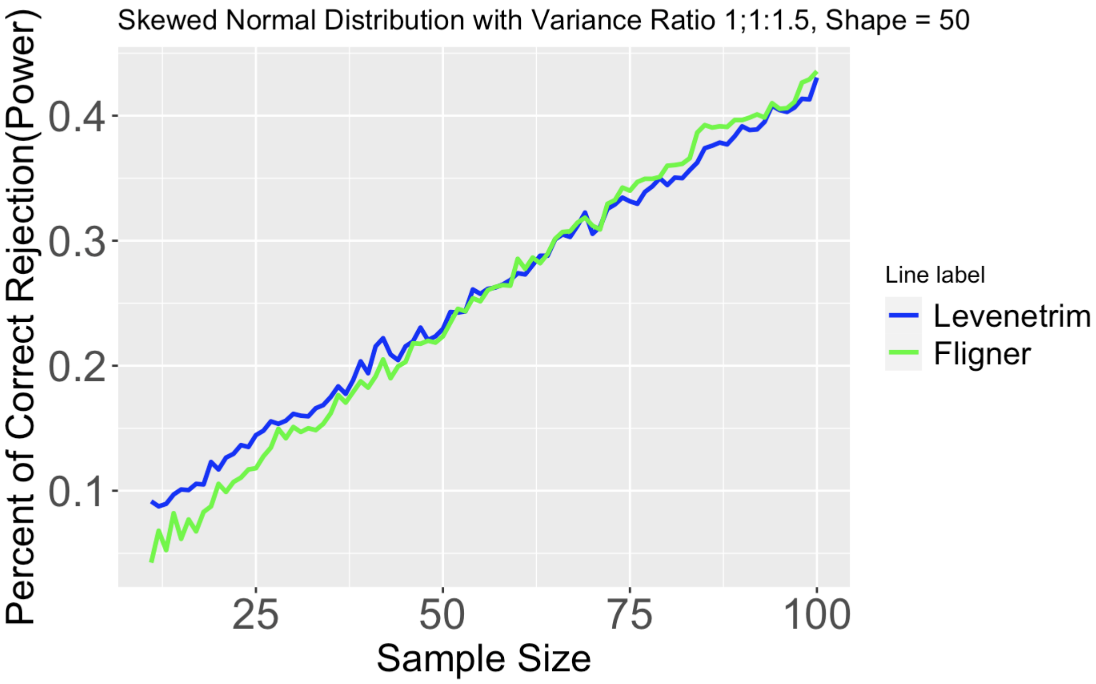

Figure 32: Proportion of correct rejection, or power, of different tests out of 2000 runs. Three skewed normal distributions(shape = 50) have same mean, but differ in variance ratio(1:1:1.5). Sample size ranges from 11 to 100.

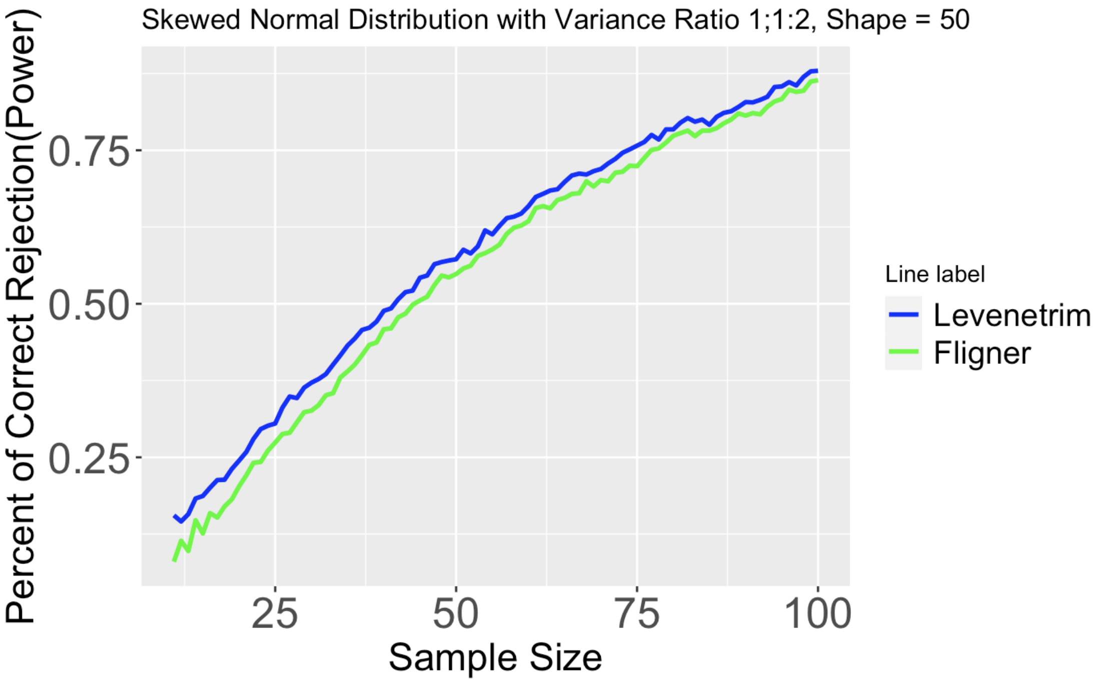

Figure 33: Proportion of correct rejection, or power, of different tests out of 2000 runs. Three skewed normal distributions(shape = 50) have same mean, but differ in variance ratio(1:1:2). Sample size ranges from 11 to 100.

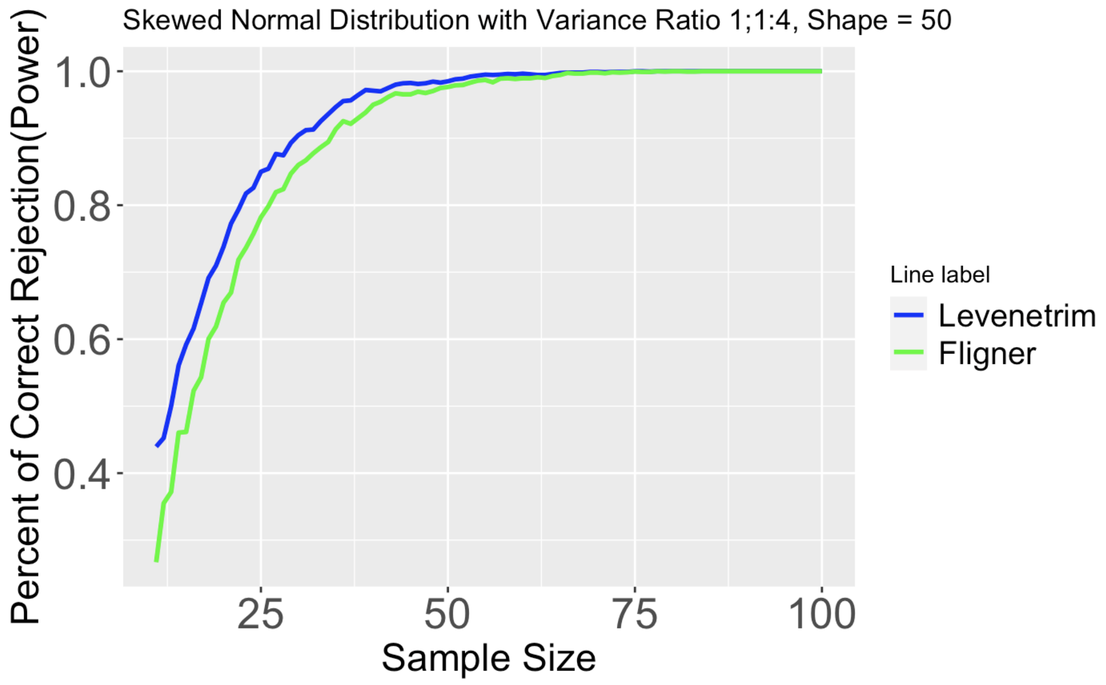

Figure 34: Proportion of correct rejection, or power, of different tests out of 2000 runs. Three skewed normal distributions(shape = 50) have same mean, but differ in variance ratio(1:1:4). Sample size ranges from 11 to 100.

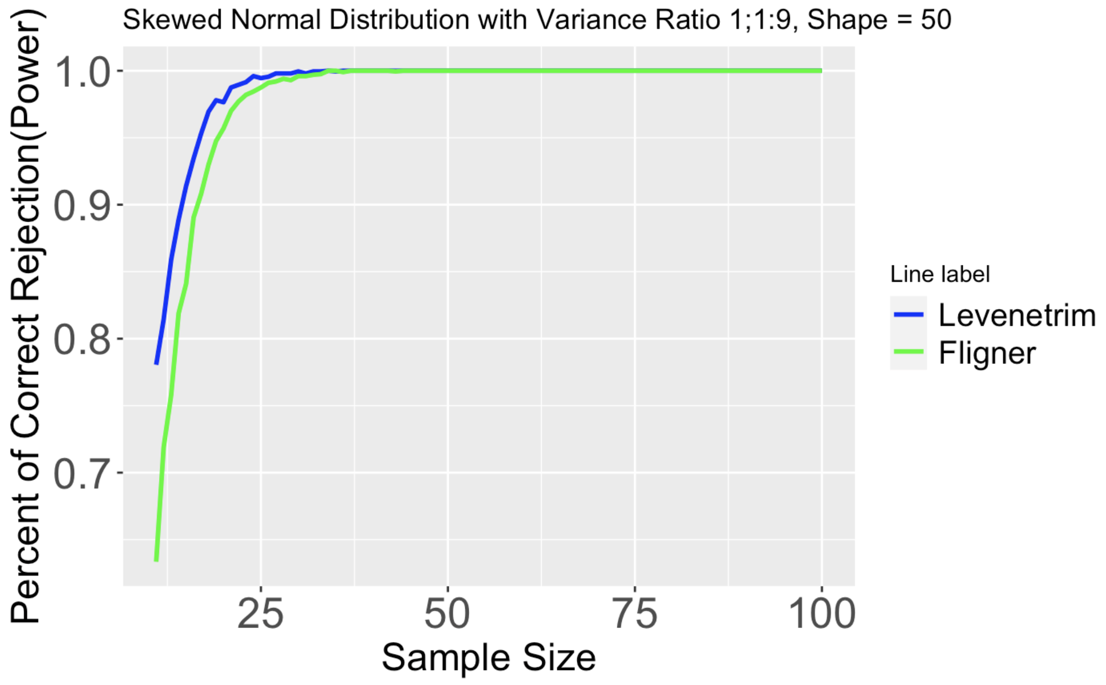

Figure 35: Proportion of correct rejection, or power, of different tests out of 2000 runs. Three skewed normal distributions(shape = 50) have same mean, but differ in variance ratio(1:1:9). Sample size ranges from 11 to 100.

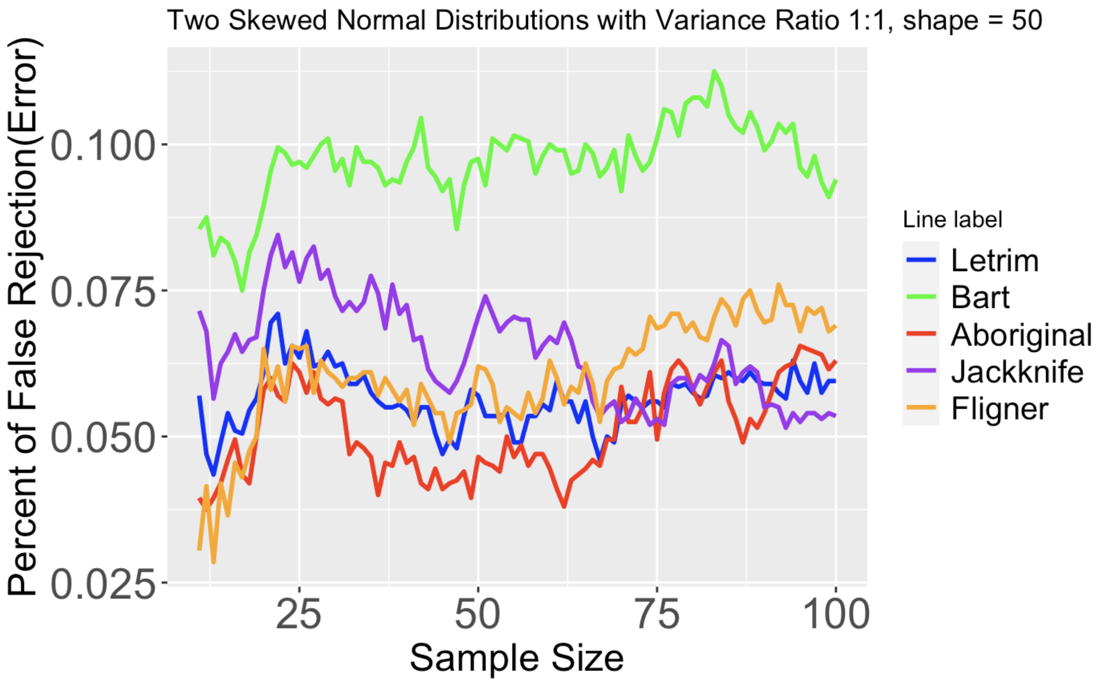

Figure 36: Proportion of false rejection, or error, of different tests out of 2000 runs. Two skewed normal distributions with same mean and same variance, shape = 50. Sample size ranges from 11 to 100.

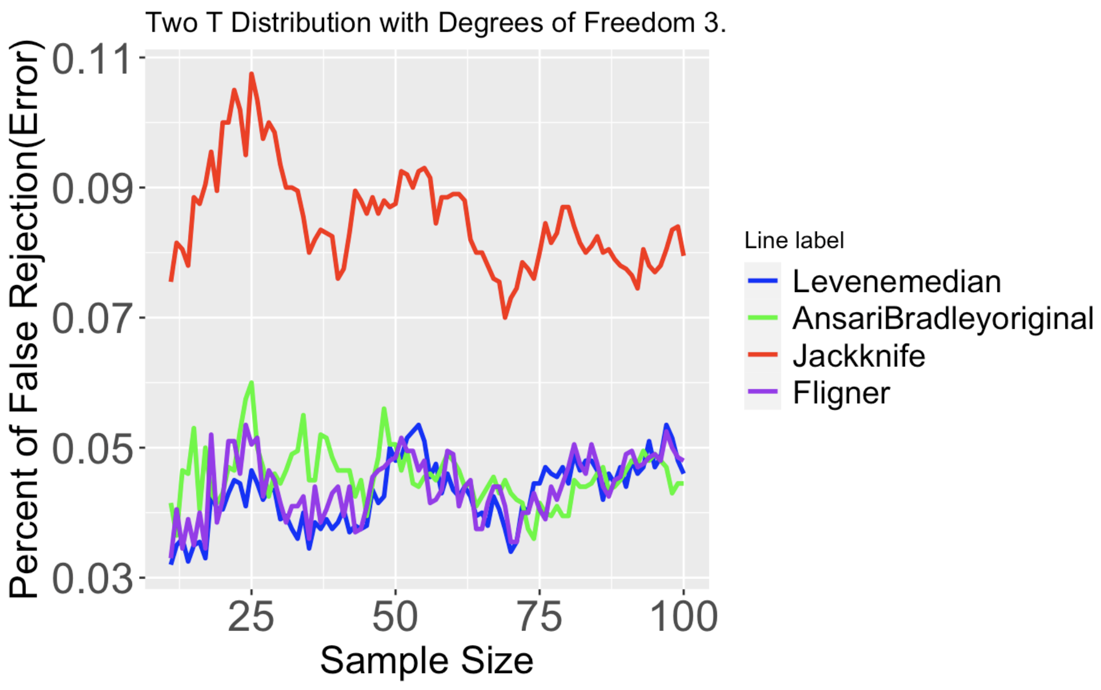

Figure 37: Proportion of false rejection, or error, of different tests out of 2000 runs. Two T distributions with degrees of freedom 3. Sample size ranges from 11 to 100.

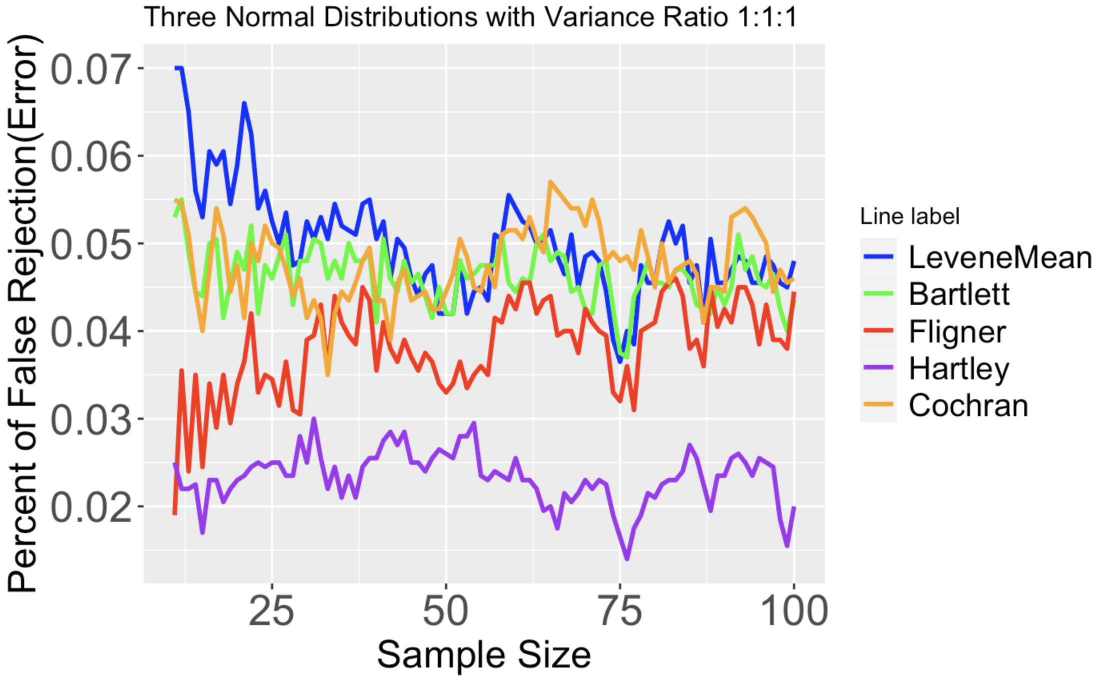

Figure 38: Proportion of false rejection, or error, of different tests out of 2000 runs. Three normal distributions with same mean and same variance. Sample size ranges from 11 to 100.

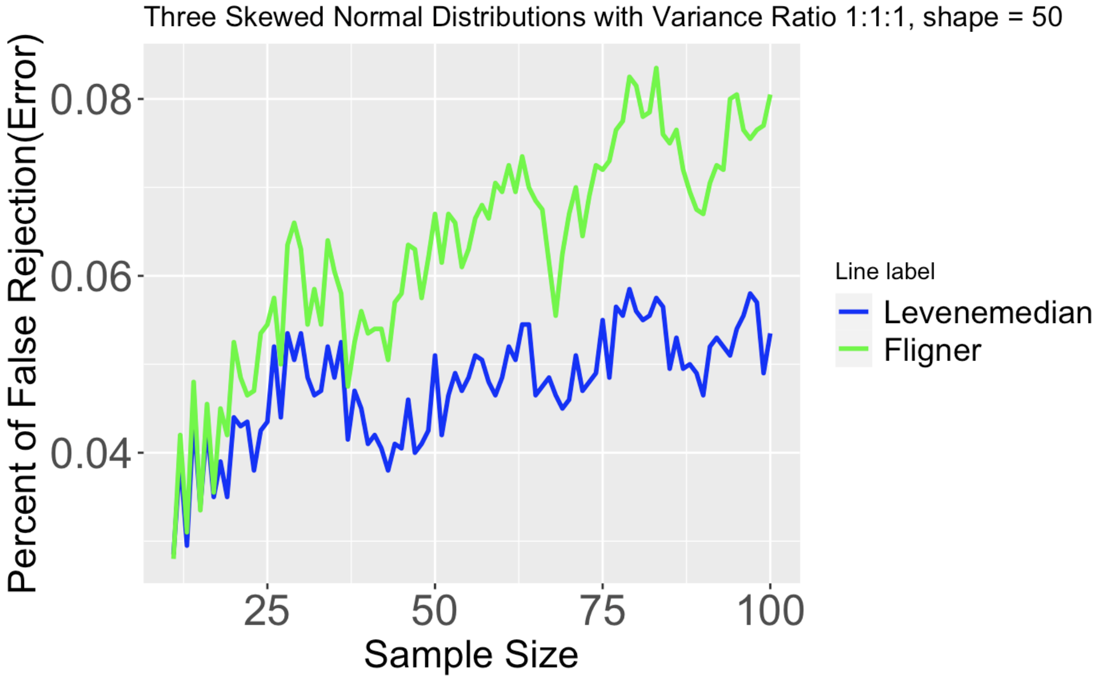

Figure 39: Proportion of false rejection, or error, of different tests out of 2000 runs. Three skewed normal distributions with same mean and same variance, shape = 50. Sample size ranges from 11 to 100.

Name of the Test	P-value
F-test	0.0001
Ansari-Bradley test	0.0008
Jackknife test	0.0001
Levene's test	0.0007
Bartlett's test	0.0006

Figure 40: P-values resulted from different variance tests. The two groups for comparison are skintot scores recorded in the first visit versus the 5th visit.
